# Context-driven mHealth solutions to improve maternal healthcare uptake in Ethiopia: Integrating stakeholder preferences and expert priorities using qualitative and nominal group techniques

**DOI:** 10.1371/journal.pdig.0001817

**Published:** 2026-11-05

**Authors:** Manaye Yihune Teshale, Mark Spigt, Yalemzer Agegnehu, Eden Hailegiorgis, Rik Crutzen, Sarah E. Stutterheim

**Affiliations:** 1 School of Public Health, Arba Minch University, Arba Minch, Ethiopia; 2 Department of Health Promotion, CAPHRI Care and Public Health Research Institute, Maastricht University, Maastricht, Netherlands; 3 Department of Family Medicine, CAPHRI Care and Public Health Research Institute, Maastricht University, Maastricht, Netherlands; Iran University of Medical Sciences, IRAN, ISLAMIC REPUBLIC OF

## Abstract

Maternal continuum of care during pregnancy, childbirth, and the postpartum period is essential for reducing maternal and child morbidity and mortality, yet access remains limited in low- and middle-income countries. In Ethiopia, only about one-quarter of women complete the continuum of maternal healthcare. Although mobile health interventions have shown promise in improving access in resource-constrained settings, successful implementation depends on cultural appropriateness and alignment with local contexts. This study explored context-driven mobile health solutions and implementation challenges in Ethiopia by integrating stakeholder preferences with expert priorities. We conducted a qualitative exploratory and consensus-prioritization study involving interviews and focus group discussions with 72 perinatal women, male partners, and key informants, complemented by a nominal group technique workshop with 14 multidisciplinary experts. Data were collected in August 2025 using semi-structured guides in Gamoththo and Amharic. Qualitative data were analyzed using reflexive thematic analysis in NVivo, and descriptive statistics summarized expert rankings. Preferred mobile health components included video-based maternal health education, short message reminders with follow-up calls, and engagement of perinatal women with their spouses or support persons. Community health workers were identified as key to developing and sharing educational videos at women’s gatherings. Participants preferred reminders between sunrise and midday on the day before appointments. Expert rankings prioritized reminders with follow-up calls (93.1%), engagement of perinatal women and their support persons (91.7%), and reminder timing between sunrise and midday (95.6%). Integrating stakeholder perspectives with expert rankings showed consensus on locally acceptable mobile health components and identified implementation challenges, including limited device access, poor network coverage, and low digital literacy. These findings provide a foundation for the co-design, feasibility testing, and pilot evaluation of contextually tailored mobile health interventions to improve maternal healthcare. Future implementation should address barriers related to device access, digital literacy, network connectivity, cost, and collaboration with telecommunications providers.

## Author summary

Many women in Ethiopia do not receive all the healthcare services they need during pregnancy, childbirth, and the period after birth. Mobile health interventions, such as text message reminders and educational videos, may help women access these services. However, these interventions need to reflect local cultures, preferences, and practical circumstances to be effective. We explored how mobile health could better support maternal healthcare in Ethiopia. We collected views from 72 pregnant or postpartum women, male partners, and other key informants through interviews and group discussions. We also consulted 14 experts to identify and prioritize preferred approaches. Participants favored educational videos developed and shared by community health workers, text message reminders followed by telephone calls, and involving women’s spouses or other chosen support persons. They preferred receiving appointment reminders between sunrise and midday on the day before a scheduled visit. Experts also prioritized these approaches. However, limited access to mobile devices, poor network coverage, low digital literacy, and affordability may hinder implementation. These findings provide a foundation for developing and testing mobile health interventions that reflect local needs and preferences and could help improve continued use of maternal healthcare services in Ethiopia.

## Introduction

Maternal health is a vital aspect of global health, significantly influencing the well-being of mothers and their newborns [1]. However, maternal mortality remains a pressing global challenge, with a woman dying every two minutes from preventable complications during pregnancy and childbirth [1]. Alarmingly, 94% of these deaths occur in low- and middle-income countries [2], primarily in Sub-Saharan Africa (SSA), which alone accounts for nearly two-thirds of global maternal mortalities [3]. Over the past two decades, Ethiopia has made remarkable strides in improving maternal and child health. According to modeled estimates from the World Health Organization and the United Nations maternal mortality estimation inter-agency group, the ratio declined from approximately 953 deaths per 100,000 live births in 2000–267 deaths per 100,000 live births in 2020 [1]. Similarly, early neonatal mortality declined from 34 to 27 per 1000 live births during the period 2000–2019 [4]. Despite notable progress, Ethiopia still accounts for 3.6% of global maternal deaths, underlining the need for targeted interventions [1].

A vital strategy for tackling the challenges of maternal health is ensuring the maternal continuum of care is in place, which provides comprehensive healthcare throughout pregnancy, childbirth, and the postpartum period [5]. In this study, the continuum of care is described using the conventional indicator of at least four antenatal care (ANC) visits, skilled delivery care (SDC), and postnatal care (PNC) within six weeks after delivery [5], consistent with earlier Demographic and Health Survey (DHS)-based measures. However, the World Health Organization currently recommends eight ANC contacts to improve maternal and newborn health outcomes [6]. Evidence suggests that effective implementation of the continuum of care can reduce neonatal and perinatal mortality; however, the impact on maternal mortality remains less conclusive and requires further investigation [7]. Despite this, access to maternal care in SSA remains a significant problem, with only 25% of women receiving the essential services they need [8]. According to the 2019 Ethiopia Mini DHS, 74% of women with a live birth in the five years preceding the survey received ANC, 48% of live births during the same period occurred in health facilities, and 34% of women with a live birth in the two years preceding the survey received a PNC check within two days after delivery [9]. These disparities highlight the need for targeted efforts to maintain care engagement beyond ANC, increase SDC, and ensure comprehensive PNC, ultimately closing gaps in maternal care.

To improve maternal health service utilization and outcomes, Ethiopia has implemented a comprehensive three-tiered health system anchored by the community-based Health Extension Program, aiming to enhance health knowledge, develop essential skills, and improve access to primary care services. Furthermore, the Health System Transformation Plan (HSTP-II, 2020–2025) aimed to improve population health by accelerating progress toward universal health coverage, strengthening community resilience during health emergencies, reducing home deliveries, and enhancing the responsiveness of the health system to community needs. As the implementation period of HSTP-II has concluded, evidence from the present study can help evaluate progress toward these objectives, identify persistent gaps in institutional delivery service utilization, and inform the design and prioritization of subsequent national health strategies and maternal health interventions. Under the HSTP-II, Ethiopia set ambitious targets of achieving 81% ANC, 76% SDC, and 76% PNC utilization by 2025. Despite progress in expanding maternal health services, gaps in the utilization and continuity of care remain, with high dropout rates across the maternal care continuum attributed to both demand- and supply-side barriers, including limited access to information, financial constraints, long distances to health facilities, inadequate quality of care, and sociocultural factors [8,10–12].

In light of these ambitious targets and the need to address persistent gaps in maternal care utilization, demand-side strategies such as mobile health (mHealth) have emerged as promising approaches to improve service uptake and continuity of care, particularly in resource-limited settings [13,14]. By using mobile technologies such as phones, tablets, and wearables, mHealth has the potential to improve access to maternal care, facilitates communication between women and healthcare providers, and support women’s engagement in care [15,16]. Previous studies have shown that mHealth can improve ANC, promote SDC, and enhance PNC [17–20]. These interventions can be delivered through various modalities, including short message services (SMS), direct voice calls, voice messages, videos, and mobile applications, engaging diverse participants such as perinatal women, healthcare providers, social support groups, and community health workers (CHWs). The timing and delivery of messages can be customized to suit users’ needs; some may prefer communication during weekdays, while others find weekends more convenient. Messages can also be aligned with gestational stages or scheduled appointments, ensuring relevance and timeliness. However, it remains unclear which communication modalities, message content, delivery schedules, target recipients, and stakeholder roles are most acceptable, culturally appropriate, and feasible for implementation in the Ethiopian context.

To fully realize the potential of mHealth, it is essential to tailor them to the local context by ensuring reliability, feasibility, and cultural acceptability through appropriate modalities, engagement strategies, and timing [21,22]. This requires understanding not only which intervention components are preferred by perinatal women, their partners, healthcare providers, and community stakeholders, but also the knowledge, skills, and roles needed to support successful implementation [21]. In addition, identifying anticipated implementation challenges and potential strategies to address them is essential for informing future intervention development and testing [22].

The purpose of this study was to explore community preferences and apply expert prioritization to rank potential mHealth intervention options, including communication modalities, engagement approaches, timing, and stakeholder roles, while identifying potential implementation challenges through the engagement of multidisciplinary experts and key stakeholders in Ethiopia. The findings are intended to provide evidence to inform the co-design, feasibility assessment, and future evaluation of culturally appropriate mHealth interventions for maternal health in Ethiopia.

## Materials and methods

### Ethics statement

This study was conducted in accordance with the Ethiopian Health Research Ethics Guidelines and the Declaration of Helsinki. Ethical clearance was obtained from the Institutional Research Ethics Review Board of Arba Minch University, under reference number IRB/23373/25. Written informed consent was secured from all participants, ensuring they were fully informed of their rights, including the right to withdraw without any consequences. Confidentiality was upheld by assigning codes to participants for data collection and management. The research team, data collectors, and facilitators took measures to protect participants’ information by avoiding the use of personal identifiers in self-administered tools, audio recordings, and verbatim notes.

### Study design

This study employed qualitative reflexive thematic analysis to explore users’ preferences and potential implementation challenges related to mHealth solutions, focusing on modalities, engagement approaches, and timing [23,24]. Building on these insights, we then used the nominal group technique (NGT) to systematically prioritize context-specific modalities, engagement approaches, and timing, integrating both existing evidence and participant feedback [25]. To ensure adherence to qualitative reporting standards, we followed the 32-item consolidated criteria for reporting qualitative studies (COREQ) checklist [26] (S1 Appendix).

### Study setting and period

This study was conducted in the Gamo Zone, part of Ethiopia’s newly established South Ethiopia Regional State, between August 10 and August 31, 2025. Geographically, Gamo is bordered by the Dirashe Zone to the south, the South Omo Zone to the southwest, the Gofa to the west, Wolayita to the north, Lake Abaya to the northeast, and the Amaro Zone to the southeast. The administrative center, Arba Minch town, is situated approximately 505 km southwest of Addis Ababa, Ethiopia’s capital. According to the 2024/25 Gamo Zone health department report, the population totals around 1,722,949, including 459,537 women of childbearing age, of whom 58,090 were pregnant [27]. These women have access to a range of healthcare facilities, including one teaching and referral hospital, one general hospital, six primary hospitals, sixty health centers, 269 health posts, and various private clinics that provide maternal and child health services.

### Participants

The qualitative component of the study involved pregnant and postpartum women, who shared their firsthand experiences, alongside male partners, who are key decision-makers and resource providers within their families in the study area. To gain a comprehensive understanding of the local context, cultural norms, and practices influencing mHealth, we also engaged key informants such as health extension workers (HEWs), leaders of female health development armies (HDAs), and community leaders. For the NGT section, participants included maternal healthcare professionals, healthcare managers overseeing maternity services, senior maternal health researchers, and policymakers involved in program management. Additionally, we involved technology experts experienced in mHealth, representatives from non-governmental organizations (NGOs) focused on maternal health, and behavioral science experts.

### Sample size

The qualitative component of the study involved a total of 72 participants, comprising 32 pregnant women, nine postpartum women, 21 male partners, and 10 key informants such as HEWs, leaders of female HDAs, and community leaders [28]. Additionally, 14 participants took part in the NGT sessions [29], which involved two maternal healthcare professionals working in local health facilities, two healthcare managers from these facilities, two senior maternal health researchers affiliated with Arba Minch University, two policymakers or executors representing the South Ethiopia Regional Health Bureau and the Gamo Zone Health Department, two technology developers from Arba Minch University, two NGO representatives focused on maternal health in the Gamo Zone, and two behavioral science experts also from Arba Minch University.

### Sampling and recruitment

The participant recruitment process began with the purposive selection of HEWs, in collaboration with local public healthcare facilities, who assisted in identifying pregnant and postpartum women using maternal registration books within their respective catchment areas. The selected locations included Holo Kebele in Chencha Zuria District; Chano Chalba and Kolla Shelle Kebeles in Arba Minch Zuria District; Zigite Bakole Kebele in Gacho Baba District; and Arba Minch town, ensuring a diverse representation of participants. Male partners and community leaders were also recruited with the support of these HEWs to enrich the sample. For the NGT sessions, participants were purposively sampled to encompass a broad spectrum of perspectives across clinical, behavioral, policy, technological, research, and community domains. All NGT participants received an invitation letter that detailed the study’s purpose, outlined expectations, and provided information about the session’s date and time, ensuring they were fully informed and understood their participation was voluntary. Participation was voluntary for all study participants, including pregnant and postpartum women, male partners, community leaders, HEWs, leaders of the female HDAs, and NGT participants.

### Data collection

Qualitative data were collected through six focus group discussions (FGDs) involving 62 participants and ten key informant interviews (KIIs). The FGDs included two sessions each in rural and urban settings with pregnant and postpartum women, along with one session in each setting with male partners. Each FGD comprised 8–12 participants and lasted 50–90 minutes. In this study, data saturation refers to the point in data collection when new interviews yield little or no additional information relevant to the research question. Thus, the number of FGDs conducted aligns with recommendations suggesting that 4–10 groups are sufficient [30]. Additionally, ten KIIs were conducted with stakeholders, including HEWs, leaders of female HDAs, and community leaders, each lasting 25–45 minutes. This KII count was considered sufficient, as little new information was identified at the tenth interview, consistent with the literature [31].

All discussions and interviews were conducted face-to-face at locations and times convenient for the participants, using either Gamoththo (the local language) or Amharic (the official language), depending on their preferences. FGDs were held in rooms at community centers and health posts, while KIIs took place at participants’ workplaces and homes. During the FGDs, the MYT facilitated the sessions, YA recorded and took notes, and EH managed the logistics. Both the MYT and YA also conducted the KIIs. All FGDs and KIIs were audio-recorded after obtaining informed consent, with one facilitator taking field notes to capture contextual details such as body language and facial expressions, which enriched data interpretation. The facilitator and interviewers had no prior relationships with the participants, and only the authors and participants were present during data collection. All the authors had a positive attitude towards mHealth, but they strived to remain neutral in the conversations with participants.

To guide the discussions and interviews, semi-structured guides were developed based on a review of relevant literature and refined through team discussions [32,33]. These guides underwent pre-testing with similar populations outside the study areas to enhance clarity and flow. The revised guides included key questions focused on service utilization practices, access barriers, preferences for mHealth solutions, anticipated challenges, and suggestions for improvement (S2 Appendix and S3 Appendix).

We then organized an engaging one-day NGT session with experts to prioritize proposed mHealth modalities, engagement approaches, and optimal timing for enhancing maternal care. This collaborative initiative used the participants’ expertise, existing evidence, and valuable feedback within the local context. The session was facilitated by MYT, with support from YA and EH, following a structured process from preparation to consensus-building [34] (S4 Appendix). It began with a presentation summarizing our qualitative synthesis and the current literature, which highlighted barriers and facilitators to maternal care access, and tailored mHealth solutions [12,17]. Based on our review, we proposed eight mHealth modalities, which include SMS, voice calls, voice messages, videos, mobile applications, and combinations of these methods [17–20,35–37].

In addition to the mHealth modalities, we outlined five distinct engagement approaches tailored to different priority populations based on our review of existing evidence, which include perinatal women alone, perinatal women alongside healthcare providers, perinatal women with their spouses or chosen support persons, perinatal women interacting with CHWs, and a collaborative model involving perinatal women, their spouses or chosen support persons, and CHWs [20,35,38]. To maximize the effectiveness of these strategies, we also identified nine optimal timing options for engagement. These included sessions scheduled during weekday and weekend mornings, afternoons, and evenings, as well as targeted the day before appointments based on gestational weeks [35,38,39].

After ensuring that all participants fully understood the proposed solutions, we introduced a predefined set of criteria adapted from a previous study on stakeholder consensus to identify maternal health priorities in Malawi, focusing on relevance, feasibility, and acceptability [40]. Experts engaged in two rating rounds to evaluate the relevance of each option on a scale of 1–3, where a rating of 1 indicated low relevance or limited contextual fit with maternal healthcare needs and a rating of 3 indicated high relevance and strong alignment with maternal healthcare needs. For feasibility, scores reflected implementation challenges, with a rating of 1 denoting substantial difficulties and a score of 3 suggesting realistic execution within existing constraints. In terms of acceptability, participants assessed cultural suitability and comfort for the target population by assigning values based on their perceptions of trust and satisfaction [40]. Presentations were given in both Amharic and English for clarity, discussions were held in Amharic, and audio recordings were made with informed consent to capture the decision-making process and participants’ rationale. The NGT process is detailed in Fig 1.

**Fig 1:**
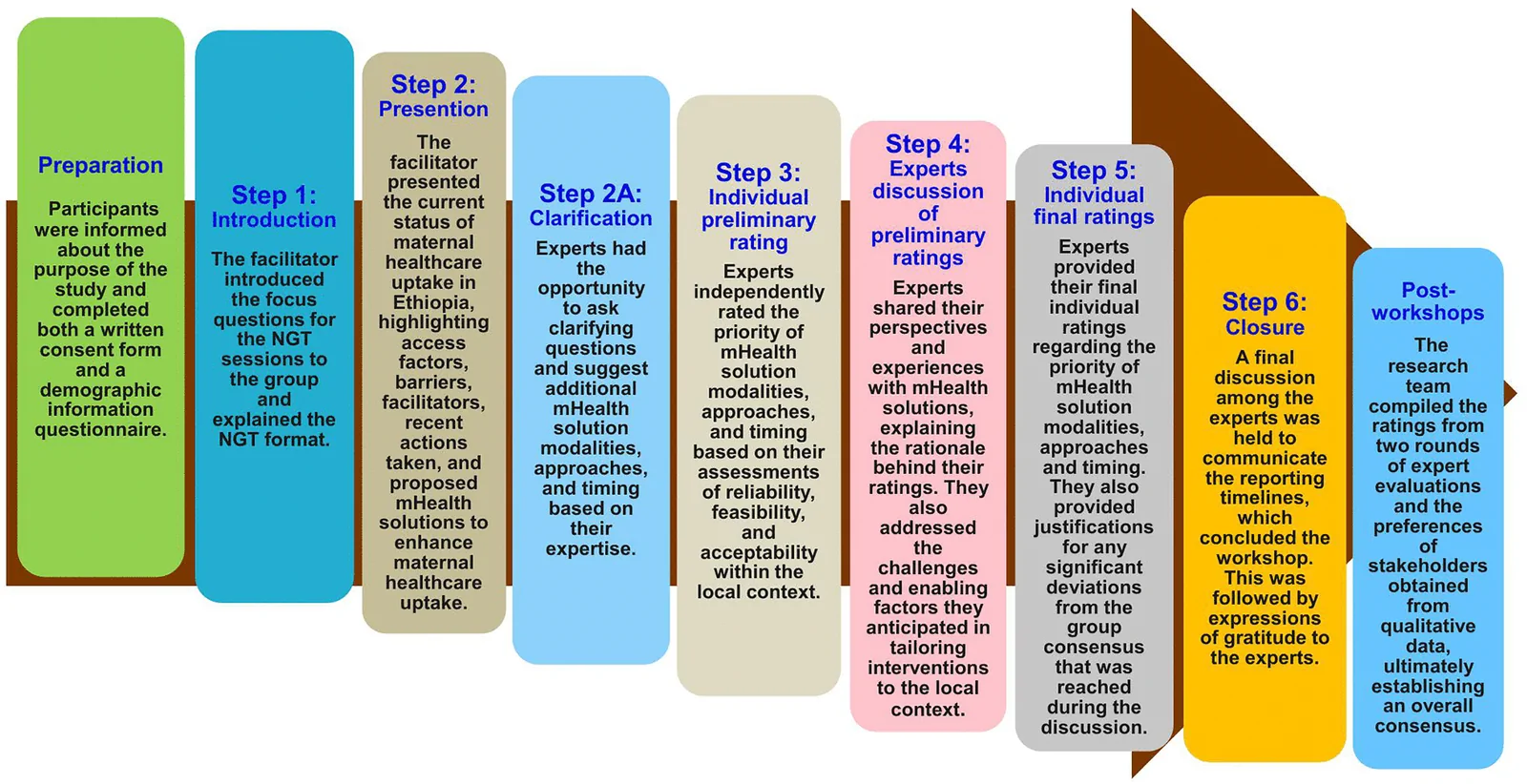
Illustration of the NGT process used for prioritizing mHealth solutions.

### Data processing and analysis

We conducted a reflexive thematic analysis to explore the qualitative data [23,24]. All audio recordings were transcribed verbatim in Gamoththo or Amharic, then translated into English by experienced bilingual researchers and imported into NVivo version 14 for data processing and analysis, along with field notes. The translated transcripts were carefully reviewed by the research team to ensure consistency and accuracy. The transcripts were not returned to participants for comments and corrections due to the time-consuming and resource-intensive logistics involved in returning them, collecting feedback, and making revisions. The analysis consisted of six key phases: familiarization with the data, initial coding, preliminary theme identification, theme refinement, final theme naming, and report production [23,24]. During the first phase, MYT and YA repeatedly reviewed the transcripts and recordings to ensure accuracy. In the second phase, MYT and YA independently coded the transcribed documents after conducting multiple readings. Following this, we prepared a codebook with definitions in a separate word document (S5 Appendix).

In the third phase, with input from EH and SS, similar codes were grouped into categories and organized into sub-themes and overarching themes through several revisions. In the fourth phase, MYT collaborated with the research team to review and refine these themes, reaching consensus after incorporating feedback and making iterative adjustments. During the fifth phase, MYT interpreted, defined, and described each theme and sub-theme, ensuring they aligned with the research questions. In the final stage, MYT compiled a comprehensive report following the COREQ. The summary of the research findings was shared with the NGT participants for member checking and validation of the prioritized findings.

Additionally, we conducted a descriptive analysis of the quantitative data collected from the NGT, which included individual preliminary ratings from Round 1 and final ratings from Round 2. The analysis followed a step-by-step approach to consolidate ratings from both rounds. First, all ratings were compiled to ensure a complete record of the items assessed by all participants in each round. All 14 experts completed the rating process in both rounds, and no ratings were missing. Next, we summed the ratings for each of the eight modality options, five approach options, and nine timing options across all experts to evaluate their relevance, feasibility, and acceptability. With 14 participants each able to award up to three points per criterion, the maximum possible score for each option per criterion was 42. Participant-level NGT ratings across modalities, engagement approaches, and timing are presented in supplementary information (S1 Table).

Aggregate scores were calculated separately for Rounds 1 and 2 by summing participants’ ratings across the three evaluation criteria. Although individual ratings were recorded on a three-point ordinal scale, the aggregated scores were used solely for descriptive consensus ranking and prioritization, consistent with established applications of the NGT, rather than for inferential statistical analyses that assume interval-level measurement [40]. The three evaluation criteria were assigned equal weight because they were considered complementary and equally important dimensions for assessing the suitability of context-specific potential mHealth interventions. No criterion was specified a priori as a limiting or dominant factor; instead, relevance, feasibility, and acceptability were evaluated collectively to provide a balanced assessment of each option. Potential feasibility constraints, such as those associated with video-based education, were explicitly considered by participants during the rating process and further explored during the structured discussions, allowing these considerations to influence the final prioritization.

To reflect the iterative nature of the NGT process, we assigned greater emphasis to Round 2 ratings (75%) than to Round 1 ratings (25%) as a study-specific analytical approach. This weighting was intended to emphasize participants’ final judgments after structured discussion, clarification of viewpoints, and reconsideration of the available evidence. The weighted composite scores were then used to rank the modality, approach, and timing options according to their relative priority. To evaluate the robustness of the prioritization, a sensitivity analysis compared the primary weighted ranking (25% Round 1 and 75% Round 2) with three alternative approaches, including Round 2-only, equal 50%/50% weighting, and median-based approaches. For the median-based approach, a participant-level total score was calculated by summing the relevance, feasibility, and acceptability ratings (range: 3–9) for each expert in each round. The median was then calculated across the 28 participant-round observations (14 experts × 2 rounds) for each modality, approach, and timing. Overall, the sensitivity analysis showed that the highest-priority modalities, approaches, and timing remained stable across the alternative aggregation methods, while some lower-ranked options showed minor changes in order. The detailed scores and rankings for each modality, engagement approach, and timing option are presented in the supplementary information (S2 Table).

To improve the interpretability of the findings, measures of response variability, including the median and interquartile range (IQR) for each option, together with the number of experts assigning the highest rating (score = 3), were summarized. These descriptive statistics complemented the aggregate scores by illustrating the distribution of expert ratings and the degree of agreement among participants. Finally, the quantitative rankings were interpreted alongside qualitative insights from the inter-round discussions, providing contextual explanations for the experts’ priorities and strengthening the interpretation of the consensus findings.

Furthermore, to develop contextually appropriate mHealth solutions, our research team integrated stakeholder preferences and expert priorities, synthesizing these perspectives to develop a comprehensive understanding of key considerations. To address discrepancies, build consensus, and obtain feedback to refine the proposed solutions, we conducted a half-day, audio-recorded consensus-building session with the participating experts. Stakeholders, including perinatal women, male partners, and key informants, were not included in the expert consensus session because their participation in the consensus process was not feasible within the study context; however, their perspectives, captured through interviews and FGDs, were considered throughout the consensus-building process.

Consensus was considered achieved when participants reached collective agreement on the prioritized modality, approach, and timing options following discussion and refinement. The final framework was developed through the consensus-building session with the participating experts, informed by the previously identified stakeholder preferences and expert NGT priorities. This collaborative process ensured that the proposed mHealth solutions reflected both stakeholder perspectives and expert judgment while remaining feasible and contextually appropriate for implementation.

### Trustworthiness

The trustworthiness of this study was upheld through several crucial activities. Experts and ethical review boards reviewed the discussion and interview guides to enhance data quality. A pre-test was conducted to evaluate the clarity, flow, and cultural sensitivity of these guides. Facilitators and interviewers utilized simple language during discussions and interviews to ensure participants’ comprehension. Additionally, all discussions were translated into English for analysis purposes, allowing for broader accessibility and ensuring that details in participants’ responses were accurately captured. Furthermore, MYT was engaged in ongoing, persistent observations of participants to gather in-depth insights. Lastly, an audit was carried out to verify the accuracy of the findings and confirm that they are well-supported by the collected data.

### Reflexivity

MYT, a male assistant professor specializing in Reproductive Health with a Master’s in Public Health, led the data collection and analysis for this study. He collaborated with YA and EH, both male researchers who hold Master’s degrees in Public Health with a focus on Health Promotion and Behavioral Science. Additionally, SS, a female researcher with a PhD and an Associate Professor in the Department of Health Promotion, along with MS, a male researcher with a PhD and an Associate Professor in the Department of Family Medicine, and RC, a male researcher with a PhD who is a Professor of Behavior Change and Technology, conducted review of the analysis and final reports.

Although our team possesses academic training and experience in qualitative research, including conducting individual interviews, facilitating FGDs, and managing and analyzing data, it is crucial to reflect on the potential biases and risks associated with our roles. The gender composition of our research team may influence perspectives and interpretations within the study. To mitigate these risks, we engaged in ongoing discussions to actively challenge each other’s viewpoints and assumptions. Furthermore, during the analysis phase, our research team sought diverse perspectives to ensure a comprehensive understanding of the data. By acknowledging these potential biases and proactively implementing strategies to address them, we aim to enhance the credibility and reliability of our research while fostering a clear understanding of the findings.

## Results

### Characteristics of the study participants

#### Participants in KIIs and FGDs.

A total of 72 participants engaged in the KIIs and FGDs for this study. The KIIs comprised 10 interviews featuring a dynamic mix of four HEWs, four female HDA leaders, and two community representatives. In parallel, we conducted six FGDs that included a diverse array of voices, including 32 pregnant women, nine postpartum women, and 21 male partners, all carefully selected from the kebeles of the Gamo Zone. Notably, 51% of participants were aged between 25 and 34 years, and 43% had achieved a primary level education. Additionally, 67% of the participants resided in rural areas, with 56% identifying as housewives (Table 1).

**Table 1:** Socio-demographic characteristics of participants in the qualitative component of the study, Gamo Zone, south Ethiopia (n = 72).

Characteristics		Number	Percent
Age	18–24	13	18
	25–34	37	51
	35–44	18	25
	≥ 45	4	6
Education	No formal education	19	26
	Primary	31	43
	Secondary	15	21
	College and above	7	10
Residence	Rural	48	67
	Urban	24	33
Occupation	Farmer	9	12
	Merchant	7	10
	Housewife	41	56
	Health extension worker	4	6
	Public servant	4	6
	Private employee	5	7
	Daily laborer	2	3

#### Participants in NGT.

All 14 invited experts participated in the nominal group discussion, achieving a 100% acceptance rate. This diverse team, with ages ranging from 28 to 49 and a mean age of 35.7, included health professionals, healthcare managers, researchers, policymakers, academicians, and representatives from NGOs. Among these distinguished individuals, two held PhDs, two served as assistant professors, and seven possessed second degrees. Notably, six participants were affiliated with academic institutions, enriching the discussion with their extensive knowledge and expertise (Table 2).

**Table 2:** Characteristics of experts involved in the NGT portion of the study in Gamo Zone, south Ethiopia (n = 14).

Experts involved in the NGT^a^	Age	Academic Rank/Credential	Working Sector
Maternal healthcare professional	29	First degree	Hospital institution
Maternal healthcare professional	49	First degree	Health center
Maternity service managers	31	Second degree	Health center
Maternity service managers	30	First degree	Hospital institution
Maternal health researcher	39	PhD^c^	Academic institution
Maternal health researcher	35	PhD	Academic institution
Policymaker or executor	48	Second degree	Regional health
Policymaker or executor	38	Second degree	Zonal health
Health information technologist	29	Assistant Professor	Academic institution
Health information technologist	36	Second degree	Academic institution
Behavioral science specialists	36	Assistant Professor	Academic institution
Behavioral science specialists	28	Second degree	Academic institution
Representative of NGOs^b^	35	Second degree	NGO
Representative of NGOs	37	Second degree	NGO

**^a^** Nominal Group Technique; ^**b**^ Non-Government Organizations; ^**c**^ Doctor of Philosophy.

### Key qualitative findings

The reflexive thematic analysis of the interview and group discussion data revealed four overarching themes which are preferred mHealth modalities, engagement approaches, timing of engagement, and perceived challenges to engagement. These themes reflect the diverse perspectives and experiences shared by participants, highlighting the complexity and details involved in mHealth implementation. They are further divided into 10 subthemes, offering a detailed exploration of underlying issues. To illustrate these key findings, relevant quotes are included below, providing richer context and deeper insights into participants’ viewpoints.

### Preferred mHealth modalities

During interviews and group discussions, participants identified three preferred modalities to enhance maternal healthcare uptake, focusing on their relevance, feasibility, and acceptability within the local context. These modalities include video-based education, SMS combined with follow-up voice calls, and direct voice calls alone. The findings identified key considerations regarding the cultural appropriateness and practical feasibility of the proposed intervention methods.

#### Video-based education.

The majority of informants and discussants preferred and emphasized the significant potential of video-based education to effectively address key prenatal topics, valuing its engaging and informative nature. They observed that many women in their communities still lack access to maternal healthcare due to limited awareness, misconceptions, and insufficient understanding of available services. The approach was believed to highlight the importance of encouraging women to attend health facilities even when they feel healthy, using visual aids that are more acceptable and engaging for women with varying knowledge levels. Utilizing videos in local languages ensures that educational messages are accessible to all women, helping bridge knowledge gaps and promote consistent prenatal care. A 31-year-old pregnant woman expressed her preference for receiving maternal health information through videos in a comfortable setting, explaining;

*“…It would be advantageous to receive maternal health information through video in a comfortable setting, such as at the health post or a community center during biweekly women’s gatherings, enabling us to learn visually and audibly about maternal health” [FGD, A 31-year-old pregnant woman].*

A 33-year-old male partner from an urban area also expressed his strong preference for video-based education during a group discussion, explaining:

*“If it is in a video, it is not easily forgotten; if you watch a video showing the benefits of maternal care and the harms of not having care, you will never forget the information. Moreover, when such education is provided not only to women but also to men, it will be valuable in addressing weaknesses and strengthening individuals. If education is disseminated in this manner, I believe it will foster unity among the community and healthcare professionals” [FGD, a 33-year-old male partner].*

A 38-year-old HEW with 12 years of rural experience also expressed a strong preference for video-based maternal health education, highlighting its effectiveness and acceptance among women based on her practical experience. She stated,

*“I believe it is more effective for mothers and us to promote the use of maternal health services through videos rather than through other health services. We have chosen videos because we are currently collaborating with other NGOs to reach married women aged 15-24, using video-based health education. This approach has been very successful; the women understand the information well, and they have expressed that it would be beneficial to have access to video education before marriage and during pregnancy” [KII, A 38-year-old HEW].*

#### SMS combined with follow-up voice calls.

Informants and discussants consistently identified a combination of SMS appointment reminders and follow-up voice calls as the most appropriate mHealth strategy for supporting maternal healthcare utilization. This preference reflected the diverse circumstances of women within the community rather than a preference for a single communication modality. SMS reminders were considered useful for women who owned or had regular access to mobile phones and were able to read text messages, enabling them to remember upcoming appointments and plan accordingly.

However, participants emphasized that literacy limitations, shared phone use, and competing household responsibilities often reduced the effectiveness of SMS messages alone. Follow-up voice calls were therefore viewed as an important complementary approach, as they provided personalized reminders, clarified information, and encouraged women to attend scheduled services. Participants also noted that voice calls could facilitate communication with family members, particularly husbands or other household decision-makers, whose support often influenced women’s ability to seek maternal healthcare. A 32-year-old postpartum woman from a rural area voiced her preference for this combination approach, stating as follows:

*“I prefer a combination of short message and direct voice call to remind me of my follow-up appointment. Most mothers do not use their cell phones that much other than to talk, so I believe a short message followed by a direct voice call would probably be more acceptable and accessible for engaging all types of women. In addition, I believe it would be great if we could see with our eyes, hear with our ears, and learn about maternal health through a video at an accessible location, which, in my opinion, is inside a health post compound” [FGD, A 32-year-old postpartum woman].*

A 33-year-old HEW with six years of rural experience expressed a preference for an integrated approach to accommodate varying literacy levels, stating:

*“Approximately 50% of mothers have the ability to read and write in our area. Hence, half of them can be reached through short messages, while the other half can be contacted through direct voice calls. Some may understand short messages, but to ensure that all mothers receive the message, following up on short messages with a direct voice call is more effective” [KII, A 33-year-old HEW].*

#### Direct voice calls.

Many informants and discussants expressed a strong preference for using direct voice calls, particularly for appointment reminders and facilitating two-way communication that allows women to ask questions and receive personalized support. They noted that many women, especially in rural communities, are either unable or less inclined to read text messages, making voice calls essential for conveying crucial information. Even in households with only one mobile device, direct calls that emphasize the importance of each visit motivate women and help them make timely healthcare decisions. During a group discussion, a 33-year-old pregnant woman expressed her preference for receiving appointment reminders solely through voice calls, explaining as follows:

*“Based on our community’s experience, many women, including me, struggle to read text messages on our phones due to the overwhelming volume of messages from various sources, including Ethio Telecom. Most of us primarily use our phones for calls and lack the knowledge or experience to navigate text messages. Our inboxes fill up quickly, leading us to delete messages without reading them. However, I believe that if my husband or I were directly called with reminders about my follow-up appointments, it would significantly help me remember to visit the health facility” [FGD, A 33-year-old pregnant woman].*

A 21-year-old postpartum woman from a rural area also highlighted her preference for using direct voice calls for appointment reminders during a group discussion, attributing her choice to the type of phone she uses. She elaborated as follows:

*“I believe that most women in our community have a cell phone, even if it is not the latest model, and those who do not have one at least have access to one within their family. Personally, I use a basic button phone, which makes it difficult for me to read the numerous text messages I receive. Because of this challenge, I prefer direct voice calls…” [FGD, A 21-year-old postpartum woman].*

A 27-year-old pregnant woman highlighted that voice communication provides an opportunity for two-way interaction and further explanation, even if they were not using their phones at the time of the call. She explained her preference as follows:

*“… We are likely to notice a missed call if we are not using our phone at the time. When we do notice it, we may choose to call back and ask questions like, where are you from? Why did you call me? This gives us an opportunity to engage further, making the act of returning the call beneficial. That is why I prefer voice calls more” [FGD, A 27-year-old pregnant woman].*

### Preferred engagement approaches

Informants and discussants identified two key engagement approaches for preferred mHealth modalities to improve maternal healthcare uptake, highlighting their relevance, feasibility, and acceptability within the local context. These approaches include engaging perinatal women and their partners, and a combined strategy that includes perinatal women, CHWs, and designated community members. These tailored strategies reflect the diverse social and community dynamics, emphasizing the importance of gender inclusion, active community participation, and personalized support to effectively enhance maternal health outcomes.

#### Engaging perinatal women and their partners.

Participants in the qualitative study emphasized the importance of engaging perinatal women and their partners as a key tailored engagement approach for mHealth solutions, particularly for appointment reminders. They highlighted that involving spouses not only encourages shared responsibility but also fosters a sense of ownership over maternal health, with partners often seen as supportive and willing to assist without hesitation. During a group discussion, a 30-year-old pregnant woman emphasized her partner’s positive role in reminding her of appointments and offering support during her pregnancy:

*“I prefer appointment reminders for my maternity visits to be sent to both my husband and me. I believe this helps us both stay informed and actively engaged in my care. It ensures that my husband also shares responsibility for both my health and the well-being of our baby, and encourages him to support me” [FGD, A 30-year-old pregnant woman].*

During a group discussion, a 25-year-old pregnant woman expressed her preference, emphasizing her frustration with the potential issues that could arise if she was solely involved in designated mHealth solutions. She explained:

*“The call should be made for both of us; if it is only for me, there could be problems at home about who made the call. Also, if we [women] keep our phones in our bags or at home, we might miss the call. I prefer my husband to be called first. Another option is to have someone close to us at home who can help us call him or her as well; it is best to call both of us” [FGD, A 25-year-old pregnant woman].*

During an interview, a 38-year-old HEW with 12 years of rural experience shared her preference, emphasizing the encouraging impact husbands can have by supporting their wives with tasks and promoting shared responsibility. She stated as follows:

*“…It would be advantageous to involve both mothers and husbands in the process. By engaging them early on, husbands can provide support to their wives with tasks, making it more convenient for them to utilize the services effectively. This approach promotes shared responsibility and ensures that both partners have access to the necessary support for maternal care” [KII, A 38-year-old HEW].*

#### Involving perinatal women, CHWs, and community members.

Informants and discussants strongly preferred a combined strategy involving perinatal women, CHWs, and designated community members, such as local health educators or community figures, in the development and dissemination of maternal health educational videos. They emphasized that CHWs and community members, being highly trusted within their communities, are well-positioned to effectively convey maternal health information as they possess a deep understanding of local cultural practices and can communicate in the community’s language. A 29-year-old postpartum woman from a rural area emphasized her preference for involving community members and local HEWs in video production and dissemination as follows:

*“It would be beneficial to offer health education in video format, but I believe many people may doubt the accuracy of the information presented. Therefore, involving community members and local health extension workers in the production and dissemination of maternal health videos would greatly enhance their credibility and effectiveness” [FGD, A 29-year-old postpartum woman].*

A 36-year-old male partner from a rural area expressed his strong preference for involving community members, identified through CHWs, in video production and dissemination using language that everyone can understand. He explained:

*“I believe it would be better if community members and those close to maternal health prepared the materials using language that everyone can understand, to better attract the attention of mothers. Additionally, it would be helpful if community health workers identified a certain number of people who could assist in creating this content” [FGD, a 36-year-old male partner].*

A 39-year-old female HDA leader from a rural area emphasized the vital role of CHWs and local community members in creating and sharing educational videos, underscoring their trusted status and cultural sensitivity:

*“I trust local health extension workers are the perfect people to create and show videos about maternal health to mothers. They are trusted in our community, know our needs and culture well, which makes the information easier to understand. Also, having a local health educator or community member with maternal health expertise involved can help ensure accurate and culturally respectful content” [KII, A 39-year-old female HDA leader].*

### Preferred timing of engagement

Informants and discussants identified two optimal timings for engagement through preferred mHealth modalities to enhance maternal healthcare uptake. These timings include from sunrise to midday on the day before scheduled appointments based on gestational weeks and during routine bi-weekly gatherings of perinatal women. They emphasized that these timeframes are highly relevant, feasible, and culturally acceptable within the local context, considering them ideal for maximizing participant engagement and ensuring effective message delivery as they align closely with daily routines and practices. Using these timeframes can improve maternal health by enhancing communication and encouraging adherence to healthcare recommendations.

#### Sunrise to midday on the day before appointments.

Participants in the qualitative study expressed strong preferences regarding the optimal timing for mHealth solutions, particularly for appointment reminders. They suggested that reminders should be scheduled for the day before appointments, specifically during the window from sunrise to midday, as this aligns with their gestational age. Many participants noted that their healthcare schedules are closely tied to their pregnancy stages, and receiving a reminder the day prior would enhance their preparedness and readiness for upcoming visits. They highlighted that individuals often check their phones for calls or messages in the early morning, making this an ideal time to deliver reminders. A 24-year-old pregnant woman, during a group discussion, shared her preference for receiving appointment reminders the day before, in the early morning. She explained:

*“Most of us are homemakers, busy with many household tasks. Because we are so busy, we prefer reminders for our appointments to be sent the day before, in the early morning. This time works best with our pregnancy schedules. For example, if my appointment is tomorrow, a reminder sent this morning would help me prepare” [FGD, A 24-year-old pregnant woman].*

A 28-year-old postpartum woman from a rural area highlighted her preference during a group discussion for receiving reminders in the morning, specifically after sunrise but before noon, on the day before her scheduled visit, expressing as follows:

*“I would like reminders to come in the morning, after sunrise but before noon, on the day before my scheduled visit. This timing is best because it allows me to prepare without rushing and considers our pregnancy stage, ensuring I can plan accordingly” [FGD, A 28-year-old postpartum woman].*

During an interview, a 39-year-old urban community leader emphasized the importance of tailoring appointment reminders to the stages of pregnancy and sending them early in the morning when women are most likely to engage with their devices. He stated,

*“I think it is best to call or text women based on how far along they are in their pregnancy, especially in the early morning when they are likely checking their phones. Personalized care reminders for each stage of pregnancy help women get important information on time and remember their check-ups, making it easier for them to use health services” [KII, A 39-year-old community leader].*

#### Bi-weekly gatherings of perinatal women.

Informants and participants also expressed their preference for integrating educational maternal health videos into perinatal women’s routine bi-weekly gatherings, which take place on weekdays in easily accessible locations such as health posts or community centers. They suggested scheduling these video sessions between sunrise to midday, as this timing fosters greater attendance and engagement. Many participants emphasized that incorporating maternal health videos into their gatherings enhances their learning experience and serves as an additional incentive for them to come together as a group. A 28-year-old pregnant woman, during a group discussion, expressed her preference for incorporating videos into their biweekly meetings at the health post, stressing its importance for those who struggle with written health information. She shared,

*“If health extension workers show us health videos during our biweekly meetings at the health post, I think it will help us understand our pregnancy better and why going to health facilities during maternity is essential. Watching videos together will make learning easier using visuals and sounds. Gathering for health information would be more useful, especially since some of us may struggle to understand written health information” [FGD, A 28-year-old pregnant woman].*

During a group discussion, a 25-year-old pregnant woman shared her thoughts, stating that watching maternal health videos during their biweekly morning meetings offers a convenient and relaxed setting to absorb essential information. She explained:

*“…Watching health videos during our biweekly morning meetings, or optionally on Sundays or holidays, works best for us. These times are convenient because we already gather at the health post every two weeks. Incorporating videos into our routine gives us more reasons to come together and learn as a group” [FGD, A 25-year-old pregnant woman].*

During an interview, a 32-year-old female HDA leader from a rural area expressed her preference for the optimal timing to show educational videos on various perinatal topics during women’s routine gatherings. She stated as follows:

*“In our community, pregnant women gather every two weeks at the community center. I believe it would be helpful to show educational videos during these regular meetings. These videos can educate them about pregnancy and labor warning signs, ensuring safe delivery at the health facility, the benefits of seeking prenatal care, and other important perinatal topics. This approach will ensure that they receive respectful care and crucial information. I think including these videos in their routine gatherings is good” [KII, A 32-year-old female HDA leader].*

### Preference variation by participant characteristics

#### Preferences by literacy level and mobile phone access.

Participants consistently indicated that preferences for mHealth modalities varied according to literacy levels and access to mobile phones. Women who were able to read text messages and had regular access to a mobile phone considered SMS appointment reminders useful for planning and remembering scheduled maternal healthcare visits. In contrast, participants noted that women with limited literacy or those relying on shared household phones often faced difficulties reading or accessing SMS messages independently. For these women, direct voice calls were considered more appropriate because they conveyed information clearly, provided opportunities for two-way communication, and enabled women to ask questions or seek clarification. Consequently, participants preferred combining SMS reminders with follow-up voice calls to accommodate differences in literacy and phone accessibility.

#### Preferences by rural context and household decision-making.

Participants, particularly those familiar with rural communities, emphasized that contextual factors might influence the acceptability and feasibility of different mHealth modalities. They explained that women living in rural areas often experience lower literacy levels, limited access to smartphones and reliable internet services, and greater dependence on shared household phones, making voice calls more practical than text-based communication alone. Participants also highlighted the influence of household decision-making on maternal healthcare utilization, noting that husbands and other family members frequently play an important role in decisions about seeking care. Accordingly, voice calls were perceived as valuable because they could engage both women and influential family members, fostering shared decision-making and encouraging timely attendance at maternal healthcare services.

#### Preferences by participant type and implementation context.

While participants recognized the importance of mHealth interventions, their specific preferences varied based on their roles and experiences. Perinatal women primarily valued video-based education for its engaging format, local language options, and ability to improve comprehension regardless of educational background. They favored integrating these videos into routine bi-weekly gatherings at health posts or community centers to facilitate collective learning. CHWs and other community representatives likewise supported the use of locally developed videos but emphasized that they should serve as complementary educational tools rather than stand-alone communication strategies. Across all participant groups, there was a shared preference for appointment reminders to be delivered the day before scheduled visits, between sunrise and midday, when women were most likely to have access to their phones.

### Perceived challenges to engagement

The interview and group discussion data also revealed a theme that shed light on the perceived challenges surrounding the adoption and utilization of preferred mHealth solutions in maternal care. This theme underscores the primary barriers that impede the successful implementation of these solutions aimed at enhancing maternal healthcare. The challenges identified grouped into three key categories which are limited access to devices, connectivity issues, and concerns regarding digital literacy. These obstacles hinder accessibility to mHealth interventions and contribute to a reduction in trust towards these innovative healthcare approaches.

#### Limited access to devices.

Qualitative data from informants and discussants highlighted limited access to devices as a major challenge in implementing effective mHealth solutions for maternal care. Many perinatal women lack mobile phones or compatible devices, which often cannot store multiple SMS messages, leading to notifications about insufficient storage after just a few messages. Additionally, limited access to charging facilities and electricity, especially in rural areas, was frequently cited as a potential barrier that could hinder consistent use. A 31-year-old pregnant woman from a rural area emphasized these concerns during a group discussion:

*“…I believe adding mobile health devices in our village might be difficult. Sometimes, there is no power to charge our phones, and some mothers do not have phones because they cannot afford them. Also, even if we do have phones, they cannot store many messages, so we might miss important health tips. These issues might make it hard to get the health information we need consistently through calls or texts” [FGD, A 31-year-old pregnant woman].*

A 34-year-old male partner from a rural area also highlighted limited access to devices as a major challenge. He suggested that using both the husband’s and wife’s phones could help address this issue and improve maternal healthcare uptake. He explained:

*“Yes, some obstacles will not go away. For example, being a rural area, there is often a lack of electricity to charge our mobile phones; …. The main thing is that some people do not have phones, and others have limitations in using their phones. Luckily, if you take both of our phones [the husband’s and the wife’s], if one of us is having trouble, the other one will work; I don’t think it will cause any problems” [FGD, A 34-year-old male partner].*

#### Connectivity issues.

Informants and discussants highlighted that limited mobile network coverage, especially in rural areas, remains a big challenge in implementing effective mHealth solutions for maternal care. Participants repeatedly noted that mobile network connectivity in many rural regions depends heavily on electricity; when power outages occur, the entire network can go down. This disruption could severely hinder the dissemination of maternal health information and reminders through their phone. Although connectivity challenges were discussed extensively, concerns related to the cost of mobile data, airtime, or phone use were not commonly raised during the interviews and FGDs. A 41-year-old HEW with eight years of experience working in a rural area emphasized:

*“The current issues are the mobile network coverage and reliability of electricity. When the power goes out, the entire network is down, which severely impacts our ability to disseminate maternal health information and appointment reminders over the phone. This disruption causes delays in communication and makes it difficult for mothers who need immediate support or guidance to get important health messages” [KII, A 41-year-old HEW].*

A 29-year-old postpartum woman from a rural area emphasized the limited mobile network coverage as a potential challenge. She explained that even if they take their phone to town to charge it, they still face difficulties due to poor signal reliability. During a group discussion, she shared her concerns, stating:

*“Thank you. For me, the biggest challenge in accessing pregnancy-related health information over the phone is the mobile network coverage. Even if we take our phone to town and charge it, we occasionally face network challenges when trying to receive and make calls” [FGD, A 29-year-old postpartum woman].*

A 38-year-old HEW with 12 years of experience in a rural area also highlighted during an interview that poor mobile network could pose a challenge. She also suggested that actively involving voluntary messengers, community volunteers who work closely with mothers, as the primary means of delivering health information and support in the community might help reach women who face difficulties with unreliable signals. She explained:

*“…Using mobile health in our rural Kebele is particularly challenging mainly because of poor mobile network coverage. I believe these issues can be addressed by actively involving voluntary messengers, community volunteers who work closely with mothers. These messengers are the primary means through which health information and support typically reach mothers in the community, and their involvement could help overcome some of the current obstacles” [KII, A 38-year-old HEW].*

#### Digital literacy concerns.

Digital literacy challenges, such as lack of familiarity and technical skills, could hinder the effective implementation of mHealth solutions, especially among those with limited technological experience. Many informants and discussants expressed those operating phones, such as opening and reading messages, could be confusing and challenging, often causing them to ignore important health information sent via messaging. A 45-year-old community leader from a rural area highlighted these concerns during an interview:

*“I want to discuss some issues women might face when using their mobile phones for health-related purposes. Some women may find it challenging because they are not very familiar with how to operate phones, such as opening and reading messages. This lack of familiarity and technical skill might hinder their engagement with mobile health” [KII, A 45-year-old community leader].*

During a group discussion, a 25-year-old pregnant woman from a rural area emphasized the challenge posed by a lack of knowledge and technical skills regarding technologies. She suggested that combining messages with calls would be more effective, as many people do not read messages thoroughly. She explained:

*“…Some people read messages, but many mothers do not. Just now, we were a little late for the discussion, but when our coordinator called, we gathered quickly. We were all excited and came in a hurry. Not only my phone, but also my neighbors’ phones, were involved too. I also think it’s very effective if messages and calls are combined. Most people do not read messages, so it is better if the call accompanies the message” [FGD, A 25-year-old pregnant woman].*

A 36-year-old female HDA leader from a rural area noted during an interview that most women struggle to operate phones without assistance from their children or a trusted person, due to limited knowledge and experience. She explained:

*“I believe that the majority of women struggle to operate phones without the help of their children or a chosen person close to them due to a lack of knowledge and experience. Engaging their husbands or chosen persons in sending messages or calls can assist in ensuring that the health information is received and understood, particularly for women who are unable to read messages” [KII, A 36-year-old female HDA leader].*

### Key NGT findings

#### Prioritizing of mHealth modalities.

All 14 invited experts participated in a two-round rating process to prioritize context-specific mHealth modalities aimed at improving maternal healthcare. In Round 1, experts independently evaluated eight proposed modalities, derived from stakeholders’ feedback and existing evidence, based on their relevance, feasibility, and acceptability, which resulted in initial rankings. The top three modalities identified were combined SMS with direct voice calls, direct voice calls, and SMS. In Round 2, after engaging in discussions and revising scores, the rankings experienced slight adjustments; however, combined SMS with voice calls and direct voice calls retained their top positions, while combined SMS with voice messages climbed to third place.

The final priority recommendations, derived from the weighted average ratings across both rounds, established the top three mHealth modalities as follows: first priority: combined SMS with direct voice calls (93.1%); second priority: direct voice calls (84.4%); third priority: combined SMS with voice messages (71.3%). This analysis underscored that while Videos and Mobile Apps were among the least favored options, the combined strategies utilizing SMS and voice communication hold significant promise for enhancing maternal healthcare uptake in the local context (Table 3).

**Table 3:** Expert ratings of priority for evidence-based mHealth modalities.

mHealth modalities	Number of Participants	Round 1 rating				Round 2 rating				Weighted Average Score (Rounds 1 and 2; Maximum = 126) N (%)	Priority rank
		R^d^	F^e^	A^f^	T^g^	R	F	A	T		
SMS^h^	14	23	34	31	88	23	33	27	83	84.3 (66.9)	6^th^
Voice messages	14	33	25	26	84	35	24	28	87	86.3 (68.5)	4^th^
Direct voice calls	14	34	32	38	104	36	33	38	107	106.3 (84.4)	2^nd^
Video messages	14	29	18	26	73	27	24	31	82	79.8 (63.3)	7^th^
Mobile Apps	14	26	15	21	62	25	16	18	59	59.8 (47.5)	8^th^
Combined SMS with voice messages	14	31	25	30	86	34	24	33	91	89.8 (71.3)	3^rd^
Combined SMS with video messages	14	33	18	28	79	36	22	30	88	85.8 (68.1)	5^th^
Combined SMS with direct voice calls	14	39	36	40	115	40	37	41	118	117.3 (93.1)	1^st^

**^d^** Relevance; ^**e**^ Feasibility; ^**f**^ Acceptability; ^**g**^ Total; ^**h**^ Short Message Services; Weighted average score: The final score was calculated by assigning 25% weight to Round 1 and 75% weight to Round 2, with a maximum possible score of 126. The percentage in parentheses represents the weighted average score as a percentage of the maximum possible score (weighted average score ÷ 126 × 100); higher scores indicate greater priority.

#### Expert ratings variability across mHealth modalities.

Across both rounds, expert ratings were highest for interactive, phone call–based delivery modalities, with the combination of SMS and direct voice calls receiving the most consistent ratings. By Round 2, this modality achieved the maximum median score across all three evaluation criteria (median = 3.0; IQR = 0.00). Specifically, 12 (85.7%) experts assigned the highest possible score (three) for relevance, while 13 (92.9%) experts awarded the highest score for acceptability. In contrast, smartphone-dependent digital tools, particularly mobile apps, received the lowest ratings and exhibited the greatest variability. For feasibility, none of the 14 experts assigned the highest possible score in either round (median = 1.0; IQR = 0.00) (Table 4).

**Table 4:** Expert ratings variability across mHealth modalities.

mHealth modalities	Criteria	Round	Number of Participants	Median Score	IQR^i^	Participants Scoring 3; N (%)
SMS^h^	R^d^	1	14	2.0	1.00	0 (0.0)
		2	14	2.0	1.00	1 (7.1)
	F^e^	1	14	3.0	1.00	8 (57.1)
		2	14	3.0	1.00	8 (57.1)
	A^f^	1	14	2.0	0.00	3 (21.4)
		2	14	2.0	1.75	4 (28.6)
Voice messages	R	1	14	2.0	1.00	6 (42.9)
		2	14	2.5	1.00	7 (50.0)
	F	1	14	2.0	1.00	3 (21.4)
		2	14	2.0	1.00	2 (14.3)
	A	1	14	2.0	0.75	3 (21.4)
		2	14	2.0	0.75	3 (21.4)
Direct voice calls	R	1	14	3.0	0.00	9 (64.3)
		2	14	3.0	0.00	11 (78.6)
	F	1	14	2.5	1.00	7 (50.0)
		2	14	2.5	1.00	7 (50.0)
	A	1	14	3.0	0.00	11 (78.6)
		2	14	3.0	0.00	12 (85.7)
Video messages	R	1	14	2.0	1.75	5 (35.7)
		2	14	2.0	1.75	4 (28.6)
	F	1	14	1.0	0.75	0 (0.0)
		2	14	1.5	1.00	3 (21.4)
	A	1	14	2.0	1.00	3 (21.4)
		2	14	2.0	1.00	5 (35.7)
Mobile Apps	R	1	14	1.5	2.00	5 (35.7)
		2	14	1.5	1.75	4 (28.6)
	F	1	14	1.0	0.00	0 (0.0)
		2	14	1.0	0.00	0 (0.0)
	A	1	14	1.5	1.00	0 (0.0)
		2	14	1.0	0.75	0 (0.0)
Combined SMS with voice messages	R	1	14	2.0	0.75	4 (28.6)
		2	14	2.0	1.00	6 (42.9)
	F	1	14	2.0	0.00	1 (7.1)
		2	14	2.0	0.75	1 (7.1)
	A	1	14	2.0	0.75	4 (28.6)
		2	14	2.0	1.00	5 (35.7)
Combined SMS with video messages	R	1	14	2.0	1.00	6 (42.9)
		2	14	3.0	1.00	8 (57.1)
	F	1	14	1.0	0.75	0 (0.0)
		2	14	2.0	0.00	0 (0.0)
	A	1	14	2.0	0.00	3 (21.4)
		2	14	2.0	0.00	3 (21.4)
Combined SMS with direct voice calls	R	1	14	3.0	0.00	12 (85.7)
		2	14	3.0	0.00	12 (85.7)
	F	1	14	3.0	0.00	11 (78.6)
		2	14	3.0	0.00	11 (78.6)
	A	1	14	3.0	0.75	12 (85.7)
		2	14	3.0	0.00	13 (92.9)

^**d**^ Relevance; ^**e**^ Feasibility; ^**f**^ Acceptability; ^**h**^ Short Message Services; ^**i**^ Interquartile Range.

A 31-year-old male maternity service manager shared insights during the inter-round discussion with experts on the numerical evaluation of the priority of combining SMS with a direct voice call, drawing from his practical experiences. He explained as follows:

*“Combining short message services with a direct voice call is an excellent approach. As mentioned earlier, individuals may not always answer their phones when called, making a combination of the two methods more effective. In our experience with immunization, we have encountered situations where individuals lose their phones or are unavailable when contacted. By integrating a short message with a direct voice call, we can overcome these obstacles. Hence, I have allocated the maximum score of three points for all criteria, including relevance, feasibility, and acceptability” [NGT, A 31-year-old male maternity service manager].*

Although video-based maternal health education was among the least favored options based on the experts’ prioritized ratings, a 39-year-old male senior researcher strongly emphasized its relevance and acceptance based on his practical experience, stating:

*“I have given the maximum score of three points for video messaging in a special situation for its relevance. Depending on the modality, it could be effective. I have conducted my PhD using video messaging. We used a PICO projector, and we used a network to gather the mothers. Trained designated leaders will play the video for the mothers. While we conducted the final acceptability assessment among the mothers, they recommended video messaging. The projector was very easy to use. The video should be customized. Social support can assist them. Using a local language and context can benefit the mothers. For feasibility, I have given a score of two points {medium score}, as they would require investment. For acceptability, I have given the maximum score of three points” [NGT, A 39-year-old male senior researcher].*

### Prioritizing whom to involve in engagement

Fourteen experts participated in a two-round rating process to prioritize individuals who should be engaged in mHealth solutions to enhance maternal health care uptake. In the first round, they independently ranked five proposed groups, identifying the top three as: perinatal women and their spouses or chosen persons, perinatal women and CHWs, and perinatal women, their spouses or chosen persons, and CHWs.

The second round involved discussions that led to revised scores, with the highest-ranked group remaining perinatal women and their spouses or chosen persons, followed by perinatal women, their spouses or chosen persons, and CHWs in second place, and perinatal women and CHWs in third. The final recommendations, based on the weighted average ratings from both rounds, highlighted the importance of inclusive strategies that engage partners or chosen support persons. The prioritized groups of individuals for mHealth solutions were: first, perinatal women and their spouses or chosen persons (91.7%); second, perinatal women, their spouses or chosen persons, and CHWs (86.7%); and third, perinatal women and CHWs (85.7%). The least favored approach was identified as only engaging perinatal women (74.0%) (Table 5).

**Table 5:** Expert ratings of priority for engagement approaches in mHealth solutions.

Engagement approaches	Number of Participants	Round 1 rating				Round 2 rating				Weighted Average Score (Rounds 1 and 2; Maximum = 126) N (%)	Priority rank
		R^d^	F^e^	A^f^	T^g^	R	F	A	T		
Only perinatal women	14	23	33	32	88	22	36	37	95	93.3 (74.0)	5^th^
Perinatal women and healthcare providers	14	34	34	35	103	34	35	37	106	105.3 (83.6)	4^th^
Perinatal women and their spouses, or chosen persons	14	41	41	38	120	40	38	36	114	115.5 (91.7)	1^st^
Perinatal women and CHWs^j^	14	38	34	39	111	37	34	36	107	108.0 (85.7)	3^rd^
Perinatal women, their spouses, or chosen persons, and CHWs	14	41	32	37	110	37	34	38	109	109.3 (86.7)	2^nd^

**^d^** Relevance; ^**e**^ Feasibility; ^**f**^ Acceptability; ^**g**^ Total; ^**j**^ Community Health Workers; Weighted average score: The final score was calculated by assigning 25% weight to Round 1 and 75% weight to Round 2, with a maximum possible score of 126. The percentage in parentheses represents the weighted average score as a percentage of the maximum possible score (weighted average score ÷ 126 × 100); higher scores indicate greater priority.

#### Expert ratings variability across engagement approaches.

Across both rounds, expert ratings were highest for engaging perinatal women along with their spouses or chosen support persons, achieving the maximum median score, and exhibiting the lowest variability across the evaluation criteria. In Round 1, 13 (92.9%) experts assigned the highest possible score (three) for both relevance and feasibility (median = 3.0; IQR = 0.00). In Round 2, 12 (85.7%) experts again assigned the highest possible score for relevance (median = 3.0; IQR = 0.00). Engaging perinatal women, their spouses or chosen support persons, and CHWs consistently received high ratings, with 13 (92.9%) experts giving the highest possible score for relevance in Round 1 (median = 3.0; IQR = 0.00). In contrast, the lowest ratings for relevance were observed when targeting only perinatal women, with 2 (14.3%) experts assigning the highest possible score in Round 1 (median = 1.5; IQR = 1.00) and only 1 (7.1%) expert assigning the highest possible score in Round 2 (median = 1.5; IQR = 1.00) (Table 6).

**Table 6:** Expert ratings variability across mHealth engagement approaches.

Engagement approaches	Criteria	Round	Number of Participants	Median Score	IQR^i^	Participants Scoring 3; N (%)
Only perinatal women	R^d^	1	14	1.5	1.00	2 (14.3)
		2	14	1.5	1.00	1 (7.1)
	F^e^	1	14	2.5	1.00	7 (50.0)
		2	14	3.0	1.00	9 (64.3)
	A^f^	1	14	2.0	1.00	6 (42.9)
		2	14	3.0	1.00	9 (64.3)
Perinatal women and healthcare providers	R	1	14	2.0	1.00	6 (42.9)
		2	14	2.0	1.00	6 (42.9)
	F	1	14	2.0	1.00	6 (42.9)
		2	14	2.5	1.00	7 (50.0)
	A	1	14	2.5	1.00	7 (50.0)
		2	14	3.0	1.00	9 (64.3)
Perinatal women and their spouses, or chosen persons	R	1	14	3.0	0.00	13 (92.9)
		2	14	3.0	0.00	12 (85.7)
	F	1	14	3.0	0.00	13 (92.9)
		2	14	3.0	0.75	10 (71.4)
	A	1	14	3.0	0.75	10 (71.4)
		2	14	3.0	1.00	9 (64.3)
Perinatal women and CHWs^j^	R	1	14	3.0	0.75	10 (71.4)
		2	14	3.0	1.00	9 (64.3)
	F	1	14	2.0	1.00	6 (42.9)
		2	14	2.0	1.00	6 (42.9)
	A	1	14	3.0	0.00	11 (78.6)
		2	14	3.0	1.00	9 (64.3)
Perinatal women, their spouses, or chosen persons, and CHWs	R	1	14	3.0	0.00	13 (92.9)
		2	14	3.0	1.00	9 (64.3)
	F	1	14	2.0	1.00	6 (42.9)
		2	14	2.5	1.00	7 (50.0)
	A	1	14	3.0	0.75	10 (71.4)
		2	14	3.0	0.75	10 (71.4)

**^d^** Relevance; ^**e**^ Feasibility; ^**f**^ Acceptability; ^**i**^ Interquartile Range; ^**j**^ Community Health Workers.

During the inter-round discussion with experts, a 29-year-old female health worker shared her perspective, emphasizing the importance of involving perinatal women, their spouses, or a chosen individual as a key tailored engagement. She stated:

*“I prefer involving perinatal women, their spouses, or a chosen individual. I believe that involving multiple individuals close to the perinatal women in appointment reminders is more beneficial. If one misses the reminder, another can step in to ensure timely care. Engaging husbands in this process enhances their sense of responsibility and ownership in supporting the women. This approach ensures timely care and fosters interactions, leading me to rate it highly for its relevance, feasibility, and acceptability” [NGT, A 29-year-old female health worker].*

A 31-year-old male health information technologist also shared his practical experience, emphasizing that integrating CHWs with perinatal women and their spouses offers an essential additional layer of reminders and support, stating:

*“I advocate for the involvement of perinatal women, their spouses, and community health workers. Based on my experience with the ‘Enat Messenger’ [mother messenger] project in northern Ethiopia, which aimed to promote facility-based childbirth, I believe that solely engaging perinatal women and their spouses may result in overlooked messages due to constant proximity. However, integrating community health workers provides an essential added layer of reminders and support” [NGT, A 31-year-old male health information technologist].*

### Prioritizing the timing of engagement

Fourteen experts participated in a two-round rating process to prioritize the optimal timing of mHealth solutions. In the first round, they evaluated nine proposed timings, identifying the top three as: the day before the appointment, from sunrise to midday, based on gestational weeks; weekday midday to sunset; and the day before the appointment, from sunset to midnight, based on gestational weeks. In the second round, discussions led to revised scores, keeping the day before the appointment, from sunrise to midday as the highest-ranked timing. It was followed by the day before the appointment, from sunset to midnight, which moved up to second place, and weekday sunrise to midday in third. The final recommendations emphasized sending reminders according to gestational age, on the day before the appointment, between sunrise and midday (95.6%) (Table 7).

**Table 7:** Expert ratings of priority for optimal timing of mHealth solutions.

Timing of mHealth solutions	Number of Participants	Round 1 rating				Round 2 rating				Weighted Average Score (Rounds 1 and 2; Maximum = 126) N (%)	Priority rank
		R^d^	F^e^	A^f^	T^g^	R	F	A	T		
Weekday Sunrise to Midday	14	31	35	28	94	33	34	30	97	96.3 (76.4)	3^rd^
Weekend Sunrise to Midday	14	22	32	32	86	24	29	28	81	82.3 (65.3)	7^th^
Weekday Midday to Sunset	14	34	35	33	102	30	31	28	89	92.3 (73.3)	5^th^
Weekend Midday to Sunset	14	28	29	31	88	28	30	32	90	89.5 (71.0)	6^th^
Weekday Sunset to Midnight	14	25	27	21	73	20	24	21	65	67.0 (53.2)	8^th^
Weekend Sunset to Midnight	14	22	24	19	65	20	23	19	62	62.8 (49.8)	9^th^
The day before the appointment, from sunrise to midday	14	39	41	39	119	41	39	41	121	120.5 (95.6)	1^st^
The day before the appointment, from Midday to Sunset	14	32	31	33	96	30	33	29	92	93.0 (73.8)	4^th^
The day before the appointment, from Sunset to Midnight	14	33	31	33	97	36	34	35	105	103.0 (81.7)	2^nd^

**^d^** Relevance; ^**e**^ Feasibility; ^**f**^ Acceptability; ^**g**^Total; Weighted average score: The final score was calculated by assigning 25% weight to Round 1 and 75% weight to Round 2, with a maximum possible score of 126. The percentage in parentheses represents the weighted average score as a percentage of the maximum possible score (weighted average score ÷ 126 × 100); higher scores indicate greater priority.

#### Expert ratings variability across mHealth delivery timing.

Across both rounds, expert ratings were highest for appointment-based timing options, particularly the day before the appointment, from sunrise to midday, which exhibited the highest ratings, greatest score stability, and lowest variability across all evaluation criteria. In Round 2, this timing option received the highest possible score from 13 (92.9%) experts for both relevance and acceptability (median = 3.0; IQR = 0.00), while 11 (78.6%) experts assigned the highest possible score for feasibility (median = 3.0; IQR = 0.00). In contrast, the lowest ratings were observed for late-evening and nighttime delivery windows on both weekdays and weekends. Weekend sunset to midnight consistently received the lowest ratings, with only 2 (14.3%) experts assigning the highest possible score for feasibility in Round 2 (median = 1.0; IQR = 1.00) (Table 8).

**Table 8:** Expert ratings variability across mHealth delivery timing.

Timing	Criteria	Round	Number of Participants	Median Score	IQR^j^	Participants Scoring 3; N (%)
Weekday Sunrise to Midday	R^d^	1	14	2.0	1.00	5 (35.7)
		2	14	2.5	1.00	7 (50.0)
	F^e^	1	14	3.0	1.00	8 (57.1)
		2	14	2.5	1.00	7 (50.0)
	A^f^	1	14	2.0	1.50	4 (28.6)
		2	14	2.0	1.75	6 (42.9)
Weekend Sunrise to Midday	R	1	14	1.5	1.00	1 (7.1)
		2	14	2.0	1.00	1 (7.1)
	F	1	14	3.0	1.75	8 (57.1)
		2	14	2.0	0.75	4 (28.6)
	A	1	14	3.0	1.75	8 (57.1)
		2	14	2.0	0.00	3 (21.4)
Weekday Midday to Sunset	R	1	14	2.0	0.75	6 (42.9)
		2	14	2.0	0.75	4 (28.6)
	F	1	14	2.5	1.00	7 (50.0)
		2	14	2.0	1.00	5 (35.7)
	A	1	14	2.0	1.00	6 (42.9)
		2	14	2.0	1.50	4 (28.6)
Weekend Midday to Sunset	R	1	14	2.0	1.50	4 (28.6)
		2	14	2.0	0.00	3 (21.4)
	F	1	14	2.0	0.75	4 (28.6)
		2	14	2.0	0.00	3 (21.4)
	A	1	14	2.0	1.00	5 (35.7)
		2	14	2.0	1.00	5 (35.7)
Weekday Sunset to Midnight	R	1	14	2.0	1.00	3 (21.4)
		2	14	1.0	1.00	0 (0.0)
	F	1	14	2.0	1.75	4 (28.6)
		2	14	2.0	1.00	2 (14.3)
	A	1	14	1.0	0.75	3 (21.4)
		2	14	1.0	1.00	2 (14.3)
Weekend Sunset to Midnight	R	1	14	1.5	1.00	1 (7.1)
		2	14	1.0	0.75	2 (14.3)
	F	1	14	2.0	1.00	2 (14.3)
		2	14	1.5	1.00	2 (14.3)
	A	1	14	1.0	1.00	0 (0.0)
		2	14	1.0	0.75	1 (7.1)
The day before the appointment, from sunrise to midday	R	1	14	3.0	0.00	11 (78.6)
		2	14	3.0	0.00	13 (92.9)
	F	1	14	3.0	0.00	13 (92.9)
		2	14	3.0	0.00	11 (78.6)
	A	1	14	3.0	0.00	11 (78.6)
		2	14	3.0	0.00	13 (92.9)
The day before the appointment, from Midday to Sunset	R	1	14	3.0	1.00	8 (57.1)
		2	14	2.0	1.00	5 (35.7)
	F	1	14	2.0	1.00	6 (42.9)
		2	14	2.5	1.00	7 (50.0)
	A	1	14	2.0	1.00	5 (35.7)
		2	14	2.0	1.75	4 (28.6)
The day before the appointment, from Sunset to Midnight	R	1	14	2.5	1.00	7 (50.0)
		2	14	3.0	1.00	9 (64.3)
	F	1	14	2.5	1.75	7 (50.0)
		2	14	2.5	1.00	7 (50.0)
	A	1	14	3.0	1.00	8 (57.1)
		2	14	3.0	0.75	10 (71.4)

**^d^** Relevance; ^**e**^ Feasibility; ^**f**^ Acceptability; ^**i**^ Interquartile Range.

During the inter-round discussion, a 38-year-old male policymaker shared his perspective on the optimal timing for engagement, particularly concerning appointment reminders. He emphasized the habitual actions and practices he had observed, stating:

*“I find it best to send appointment reminders the day before, tailored to their gestational weeks and timed from sunrise to midday. Mornings are when our minds are clear, and it is a time when many of us habitually check our phones for calls and messages. This early alert gives them a chance to prepare for their next-day care visit and share the appointment details with their families or a trusted person” [NGT, A 38-year-old male policymaker].*

During the inter-round discussion, a 28-year-old male behavioral science expert shared his perspective on the optimal timing for engagement based on the content, whether a reminder or educational message, as follows:

*“I believe the timing should be based on the content. For appointment reminders, it is best to send them the day before, tailored to the gestational week, preferably from sunrise to midday. When it comes to educational messages, I prefer weekends. I see weekends as social events that present an opportunity for addressing both interpersonal and intrapersonal barriers. Sending a message in the afternoon when families gather allows the mother to raise it as a discussion topic by saying, ‘I have received such a message.’ Personally, I find Sunday afternoons particularly effective for educational message delivery” [NGT, A 28-year-old male behavioral science expert].*

### Consensus for tailored mHealth solutions

To develop contextually appropriate mHealth solutions, our research team integrated findings from stakeholder interviews with the expert priorities generated through the NGT. Comparison of these two sources revealed a clear difference, with stakeholders consistently preferring video-based education because they perceived it as an engaging and culturally appropriate approach to improving maternal health knowledge across varying literacy levels. They suggested that locally produced videos could be delivered through existing community platforms, such as biweekly gatherings of pregnant and postpartum women at health posts or community centers, thereby reducing barriers related to individual smartphone ownership and internet access.

In contrast, experts assigned relatively low priority to standalone video messages and combined SMS with video messages, primarily because of concerns about implementation feasibility, including limited smartphone ownership, unreliable electricity and internet connectivity, data costs, and varying levels of digital literacy. Consequently, experts prioritized combined SMS with direct voice calls, direct voice calls, and combined SMS with voice messages because these modalities were considered more feasible for large-scale implementation within the Ethiopian context.

To reconcile these differing perspectives, we convened a half-day consensus-building session involving the participating experts. During the session, the main issue discussed with the participating experts was video-based maternal education, which was highly preferred by stakeholders but not prioritized by the experts due to feasibility and resource considerations. After considering the stakeholder perspectives, resource requirements, and its potential relevance and acceptability within the targeted communities, the experts agreed to retain video education in the final framework as a complementary educational component delivered through existing community-based platforms, while noting that its feasibility should be further assessed through future implementation testing.

In addition, SMS and direct voice calls remained the principal communication modalities for routine maternal health messaging because they received the highest expert priority and were judged to be the most feasible and scalable. This integrated consensus ensured that the final context-driven mHealth solutions reflected both user preferences and expert judgment while remaining culturally appropriate, implementable, and responsive to local health system realities (Fig 2).

**Fig 2:**
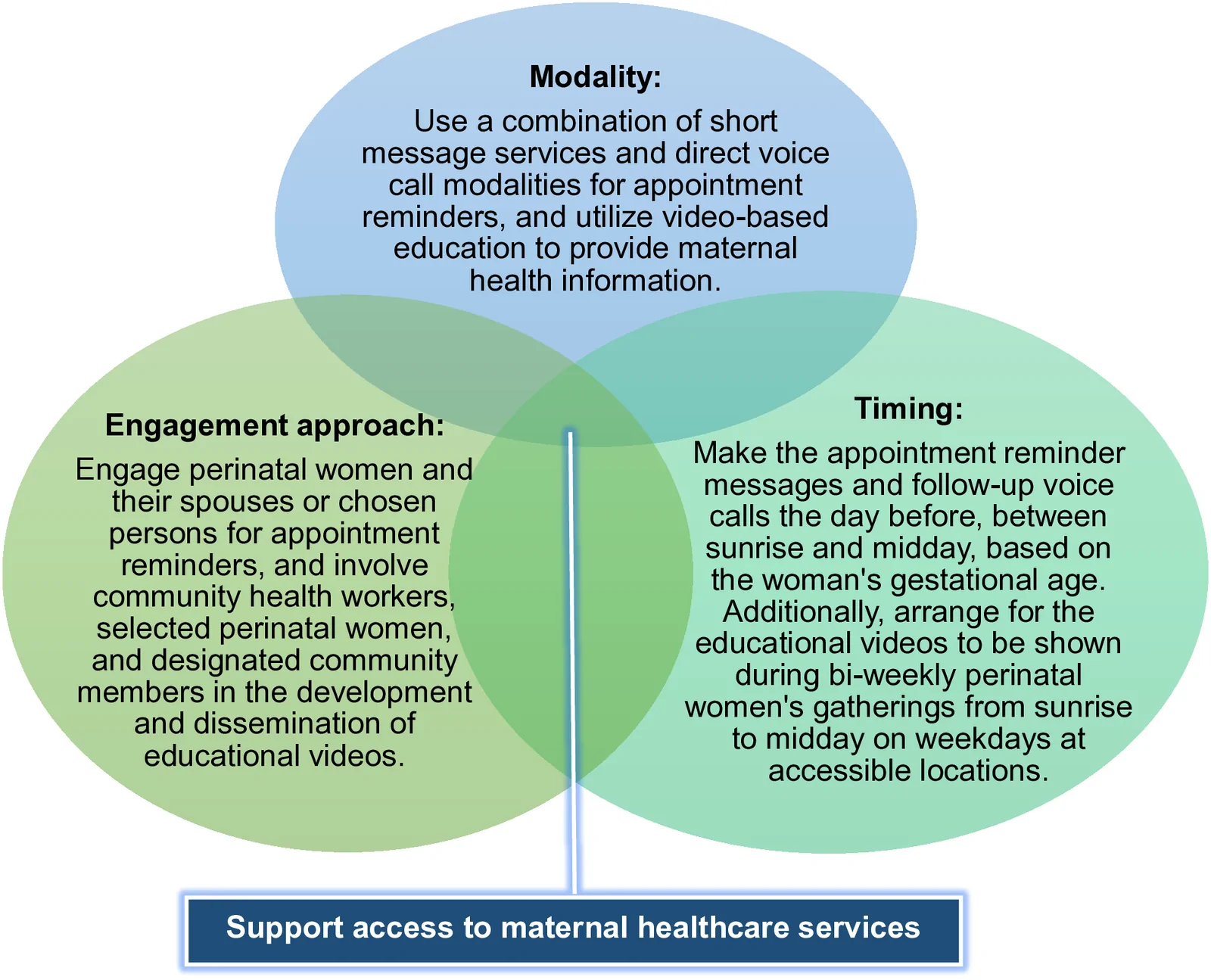
Tailored mHealth solutions based on consensus of insights from experts and stakeholders to improve maternal healthcare uptake.

## Discussion

This study provides, to our knowledge, one of the first contributions in Ethiopia to integrate the perspectives of perinatal women, male partners, CHWs, key informants, and multidisciplinary experts to inform contextually appropriate mHealth solutions for maternal healthcare. By engaging these diverse stakeholders and an expert panel, we identified culturally relevant and feasible strategies, including SMS and voice-call appointment reminders and video-based maternal health education. Participants stressed the necessity of scheduling reminders for perinatal women and their partners from sunrise to midday the day before appointments based on gestational weeks. They also highlighted the importance of CHWs in creating and sharing educational videos during bi-weekly women’s gatherings. While participants expressed strong support for a multimodal approach, they also highlighted challenges such as limited device access, poor network connectivity, and low digital literacy as critical barriers that should be addressed before wider implementation.

Participants emphasize a multimodal approach that combines simple, accessible technology with personalized communication, which was perceived as an acceptable and contextually appropriate strategy to support maternal healthcare utilization. This strategy integrates SMS reminders for quick, cost-effective appointment notifications, follow-up voice calls that foster interactive dialogue and reassurance, and engaging video-based education tailored to diverse literacy levels. Evidence from multiple studies supports the effectiveness of these modalities, with SMS reminders improving appointment adherence [18,19,37,41], follow-up voice calls enhancing message retention and addressing concerns [35], and educational videos improving maternal health knowledge and practices in rural and resource-limited settings [36,42]. Combining these methods, particularly when interactions are scheduled at culturally appropriate times, may enhance acceptability and user engagement and should be evaluated for their effectiveness in improving maternal healthcare utilization [20]. If implemented, the intervention should incorporate culturally relevant messages, convenient scheduling, locally produced educational videos, trained CHWs, and collaboration with mobile network providers.

The consensus suggests that involving key stakeholders, such as perinatal women, their spouses, and community members in mHealth interventions, may support the acceptability, contextual relevance, and implementation of maternal health interventions. Delivering appointment reminders to spouses or trusted individuals was perceived by participants as a potentially useful strategy for supporting attendance and adherence to maternal healthcare services. However, the involvement of spouses or other support persons should be implemented with careful consideration of women’s preferences, privacy, autonomy, and informed consent, as sharing health-related messages may introduce unintended risks, including partner control, confidentiality concerns, or household conflict in some settings.

Engaging CHWs in creating and sharing educational content was also perceived as an acceptable approach that may strengthen trust and ensures culturally appropriate messaging. Previous research shows that involving social support networks in mHealth positively influences maternal service use [38,43], and collaboratively developing culturally sensitive educational content enhances message effectiveness [36,44]. Nevertheless, the present study did not evaluate the effectiveness of these proposed strategies on maternal healthcare uptake. Instead, it identified stakeholder-endorsed approaches that were perceived as acceptable and contextually appropriate, and which should be evaluated in future implementation studies. Active community participation, particularly with CHWs leading content development and dissemination, may support maternal health service uptake and promotes lasting behavior change [36,45]. To optimize these efforts, it is recommended to design appointment reminders involving spouses or support persons only with the woman’s consent and according to her communication preferences, train CHWs and community members to co-create culturally tailored educational videos using participatory approaches, and combine community engagement strategies with technological solutions [46].

Scheduling appointment reminders for the day before, ideally between sunrise and midday, aligns with women’s preparedness and cultural routines, ensuring they and their families are receptive to upcoming healthcare visits. Incorporating educational videos into women’s gatherings was perceived as an acceptable approach for supporting engagement by utilizing existing social structures to deliver maternal health information. Evidence indicates that timely, personalized, and culturally sensitive reminders improve healthcare attendance and adherence [19,35,38,39,47], while community-based video education enhances maternal knowledge, attitudes, and practices [36,48]. By addressing barriers like forgetfulness through proper timing and engaging community leaders and CHWs, these strategies empower an informed and proactive maternal population. Future implementation efforts should consider scheduling personalized, culturally relevant reminders one day before appointments, incorporating educational videos into bi-weekly women’s gatherings, and regularly evaluating the timing, content, acceptability, and effectiveness of these interventions.

While experts and stakeholders perceived a multimodal mHealth approach as a feasible and acceptable strategy that may support maternal healthcare utilization, they also identified the issue of limited access to devices. In rural areas, many perinatal women lack mobile devices, which hinders their timely reception of health messages. Even for those with devices, limited storage capacity and unreliable charging facilities interrupt sustained mHealth use. These limitations in device access reduce the reach, acceptability, and potential implementation of mHealth, consistent with findings from other low-resource settings where limited device access and unstable electricity hinder utilization [20,32,49]. If left unaddressed, these barriers may contribute to the continued digital gap and could exacerbate health disparities among vulnerable women [50]. Based on stakeholder perspectives, these findings suggest several potential implementation strategies that need further evaluation, including affordable or subsidized mobile devices, utilizing family members’ devices for calls and messages, community-based initiatives like solar chargers, and partnerships with telecom providers to improve charging infrastructure. However, this study did not assess the cost, feasibility, governance requirements, provider willingness, or sustainability of these approaches. Therefore, these strategies should be considered as hypotheses for future implementation planning and evaluated in implementation research before large-scale adoption.

Participants also noted that in many rural settings, sporadic network connectivity, which is reliant on electricity, may disrupt vital health communications such as appointment reminders. This inconsistency could potentially undermine the trustworthiness of mHealth interventions among perinatal women. Similar challenges are documented in low-resource settings, indicating that network instability affects the success, adoption, and sustained use of mHealth programs [32,51]. Consequently, women living in areas with unreliable networks may experience reduced access to appointment reminders and maternal health information, potentially limiting the implementation and acceptability of mHealth interventions. To address these challenges and ensure program sustainability, policymakers should prioritize investments in expanding mobile network coverage through collaboration with telecom providers. Moreover, accompanying mHealth with voluntary health messengers to support appointment reminders and information dissemination can help reach users not reached due to connectivity issues.

Furthermore, participants revealed that limited digital literacy and unfamiliarity with mobile phone functions might present barriers to their effective engagement with mHealth interventions. Participants struggling to navigate mobile messages may lead to reduced health information use and poor adherence to recommended maternal health practices using mHealth solutions. Previous studies in low-resource settings indicate that digital literacy plays a crucial role in mHealth success, with lower technological skills preventing engagement [20,32,49,50,52]. If unaddressed, these challenges may limit the reach, acceptability, and implementation of mHealth interventions among women with low digital literacy. To address these challenges, implementing user-friendly strategies such as follow-up voice calls for interactive communication, providing digital literacy training for women and their families, establishing peer support programs, and conducting comprehensive testing before wider implementation is recommended.

### Strengths and limitations

A primary strength of this study lies in its comprehensive and inclusive approach, which integrated multiple data collection methods and engages a diverse array of stakeholders and experts. This provided a detailed understanding of decision-maker perspectives, ensured that the proposed strategies reflected community priorities, and addressed health system needs. By involving local stakeholders in the prioritization process, the study identified contextually tailored strategies, establishing a potentially replicable framework for effective mHealth solutions in similar resource-limited settings.

Several limitations should be considered. The study was conducted in the Gamo Zone of southern Ethiopia, which may limit the generalizability of the findings to other settings with different sociocultural, health system, and technological contexts. Although structured methods were used to promote equitable participation, socially desirable responses, and the influence of dominant participants during FGDs, and the NGT process may have affected the findings. In addition, transcripts were not returned to participants for member checking. Finally, this study focused on the co-design and prioritization of context-specific mHealth strategies and did not evaluate their implementation, effectiveness, feasibility, cost-effectiveness, governance requirements, or provider willingness.

Therefore, the proposed strategies should be considered stakeholder-informed approaches that require further implementation research before wider adoption. Future studies should also co-develop and evaluate culturally appropriate maternal health education videos, explore mothers’ motivations for attending and missing maternal care to optimize reminder messages, and assess the implementation outcomes and cost-effectiveness of the proposed multimodal mHealth strategies.

## Conclusions

This study identified context-driven, locally acceptable, and stakeholder-prioritized mHealth components for supporting maternal care in Ethiopia by integrating stakeholder and expert insights through a qualitative and NGT. Participants perceived a multimodal approach comprising timely SMS reminders, follow-up voice calls, and culturally tailored educational videos as an acceptable and contextually relevant strategy that may support maternal care utilization, although its effectiveness was not evaluated in this study. Community involvement, particularly the engagement of perinatal women, their spouses, and local leaders, was also perceived as important for ensuring the acceptability and contextual relevance of the proposed strategies.

Participants further prioritized delivering reminders the day before appointments during morning hours and integrating educational videos into community gatherings as contextually appropriate implementation considerations. However, infrastructural challenges such as limited device access, unreliable charging, poor network coverage, cost, and low digital literacy, were identified as important barriers that should be considered during implementation planning. Future research should evaluate the feasibility, implementation, effectiveness, and cost-effectiveness of these stakeholder-prioritized mHealth components, while also developing strategies to address infrastructural limitations and improve digital literacy to support equitable implementation.

## Supporting information

S1 Appendix Consolidated criteria for reporting qualitative studies (COREQ): 32-item checklist. (DOCX)

S2 Appendix FGD guide for pregnant, postpartum women, and male partners. (DOCX)

S3 Appendix KII guide for Key informants (HEWs, leaders of the female HDAs, and community leaders). (DOCX)

S4 Appendix NGT guide for experts’ consensus. (DOCX)

S5 Appendix Codebook. (DOCX)

S1 Table De-identified participant-level NGT ratings for mHealth solutions modalities, engagement approaches, and timing preferences across relevance (R), feasibility (F), and acceptability (A) in Rounds 1 and 2. (DOCX)

S2 Table Sensitivity analysis of mHealth modalities, engagement approaches, and timings prioritization rankings across alternative aggregation methods. (DOCX)

## Abbreviations

ANC: Antenatal Care
CHWs: Community Health Workers
COREQ: Consolidated criteria for Reporting Qualitative studies
DHS: Demographic and Health Survey
FGDs: Focus Group Discussions
HDAs: Health Development Armies
HEWs: Health Extension Workers
HSTP: Health System Transformation Plan
KIIs: Key Informant Interviews
mHealth: Mobile Health
NGO: Non-Governmental Organization
NGT: Nominal Group Technique
PNC: Postnatal Care
SDC: Skilled Delivery Care
SMS: Short Message Service
SSA: Sub-Saharan Africa
