# Supplementary material for: Context-driven mHealth solutions to improve maternal healthcare uptake in Ethiopia: Integrating stakeholder preferences and expert priorities using qualitative and nominal group techniques
Source: PLOS Digit Health. 2026 Nov 5;5(11):e0001817. doi: 10.1371/journal.pdig.0001817 (PMC13739138; doi:10.1371/journal.pdig.0001817)
Supplement: Supplementary file 1 [file pdig.0001817.s001.docx]

**S1 Appendix.** **Consolidated criteria for reporting qualitative studies (COREQ): 32-item checklist**

| **No. Item** | **Guide questions / description** | **Reported on Manuscript** | **Reported on page number** |
| --- | --- | --- | --- |
| **Domain 1: Research team and reﬂexivity** |  |  |  |
| *Personal Characteristics* |  |  |  |
| 1. Interviewer / facilitator | Which author/s conducted the inter view or focus group? | In the FGDs, the MYT served as the facilitator, the YA recorded the sessions and took notes, and EH managed the logistics needed for the sessions. Additionally, the MYT and YA conducted the KIIs. | 7 |
| 2. Credentials | What were the researcher’s credentials? E.g. PhD, MD | MYT, an assistant professor with an MPH, led the data collection and analysis for this study. He collaborated with YA and EH, both of whom also hold MPH degrees. SS, a PhD and associate professor, along with MS, a PhD and associate professor, and RC, a PhD and professor, conducted an iterative review of the analysis and final reports while overseeing the entire project. | 11 and 12 |
| 3. Occupation | What was their occupation at the time of the study? | All the authors (MYT, YA, EH, SS, MS, and RC) are researchers affiliated with academic institutions. | 11 and 12 |
| 4. Gender | Was the researcher male or female? | MYT, YA, EH, MS, and RC are male researchers, while SS is a female researcher. | 11 and 12 |
| 5. Experience and training | What experience or training did the researcher have? | All authors have academic training and extensive experience in qualitative research, including conducting individual interviews, facilitating FGDs, and managing and analyzing data. | 11 and 12 |
| ***Relationship with participants*** |  |  |  |
| 6. Relationship established | Was a relationship established prior to study commencement? | No prior relationship. | 7 |
| 7. Participant knowledge of the interviewer | What did the participants know about the researcher? E.g., personal goals, reasons for doing the research | No prior knowledge of the interviewer. | 7 |
| 8. Interviewer characteristics | What characteristics were reported about the interviewer/facilitator? E.g., Bias, assumptions, reasons and interests in the research topic | All the authors had a positive attitude towards mHealth solutions, but the authors strived to remain neutral in the conversations with participants. | 7 |
| **Domain 2: study design** |  |  |  |
| *Theoretical framework* |  |  |  |
| 9. Methodological orientation and Theory | What methodological orientation was stated to underpin the study? e.g. grounded theory, discourse analysis, ethnography, phenomenology, content analysis | Reflexive Thematic Analysis. | 10 |
| *Participant selection* |  |  |  |
| 10. Sampling | How were participants selected? e.g. purposive, convenience, consecutive, snowball | Purposive sampling. | 6 |
| 11. Method of approach | How were participants approached? e.g. face-to-face, telephone, mail, email | All discussions and interviews were conducted face-to-face, at locations and times convenient for participants, in either Gamoththo (the local language) or Amharic (the official language), based on their preferences. | 7 |
| 12. Sample size | How many participants were in the study? | We engaged 72 stakeholders, including 32 pregnant women, 9 postpartum women, 21 male partners, and 10 key informants, such as health extension workers and leaders. Additionally, we included 14 experts from various fields. | 6 |
| 13. Non-participation | How many people refused to participate or dropped out? Reasons? | All participants who completed the consent form completed the study. | 12 and 13 |
| *Setting* |  |  |  |
| 14. Setting of data collection | Where was the data collected? e.g. home, clinic, workplace | FGDs took place in a room at the community centers and health posts, while interviews were held at participants' workplaces and homes. | 7 |
| 15. Presence of non-participants | Was anyone else present besides the participants and researchers? | Only the authors were present during data collection with the participants. | 7 |
| 16. Description of sample | What are the important characteristics of the sample? e.g. demographic data, date | The details about study sample are provided in Table 1 and Table 2 | 13 and 14 |
| *Data collection* |  |  |  |
| 17. Interview guide | Were questions, prompts, guides provided by the authors? Was its pilot tested? | The research team developed the questions, prompts, and guides jointly. | 7 and, Additional File 2 and 3 |
| 18. Repeat interviews | Were repeat interviews carried out? If yes, how many? | There were no repeat interviews conducted. | 7 |
| 19. Audio/visual recording | Did the research use audio or visual recording to collect the data? | All FGDs and KIIs were audio-recorded after obtaining informed consent from the participants. | 7 |
| 20. Field notes | Were ﬁeld notes made during and/or after the interview or focus group? | Field notes were made during the focus group. | 7 |
| 21. Duration | What was the duration of the inter views or focus group? | 50-90 minutes for focus groups and 25-45 minutes for interviews. | 7 |
| 22. Data saturation | Was data saturation discussed? | In the context of this study, data saturation refers to the point in data collection when new interviews yield little or no additional information relevant to the research question. Therefore, the number of focus FGDs conducted aligned with recommendations that suggest conducting between four and ten groups is sufficient to identify a range of new issues. Additionally, the number of KIIs was considered adequate, as little new information is typically uncovered after conducting ten interviews. | 7 |
| 23. Transcripts returned | Were transcripts returned to participants for comment and/or correction? | The transcripts were not returned to participants for comments and corrections due to the time-consuming and resource-intensive logistics involved in returning them, collecting feedback, and making revisions. | 10 |
| **Domain 3: analysis and ﬁndings** |  |  |  |
| *Data analysis* |  |  |  |
| 24. Number of data coders | How many data coders coded the data? | Two authors (MYT and YA) have performed the coding independently after repeated readings of the transcribed  documents. | 10 |
| 25. Description of the coding tree | Did authors provide a description of the coding tree? | Yes. We prepared a codebook with definitions in a separate word document. | 10 and, Additional File 5 |
| 26. Derivation of themes | Were themes identiﬁed in advance or derived from the data? | Themes were derived from the data. | 10 |
| 27. Software | What software, if applicable, was used to manage the data? | NVivo version 14. | 10 |
| 28. Participant checking | Did participants provide feedback on the ﬁndings? | Yes. The summary of the research findings was checked by the participants who provided relevant feedback. | 10 |
| *Reporting* |  |  |  |
| 29. Quotations presented | Were participant quotations presented to illustrate the themes/ﬁndings? Was each quotation identiﬁed? e.g. participant number | Yes. Each quotation was identified by which FGDs or KIIs the participant was from and the gender of the participant or by the profession of the interviewee. | 14 - 29 |
| 30. Data and ﬁndings consistent | Was there consistency between the data presented and the ﬁndings? | Yes. | 14 - 29 |
| 31. Clarity of major themes | Were major themes clearly presented in the ﬁndings? | Major themes are summarized in the results. | 14 |
| 32. Clarity of minor themes | Is there a description of diverse cases or discussion of minor themes? | Yes, e.g., subthemes of preferred mHealth modalities include diverse cases. | 14 - 29 |

Reference: Tong A, Sainsbury P, Craig J. Consolidated criteria for reporting qualitative research (COREQ): a 32-item checklist for interviews and focus groups. *International Journal for Quality in Health Care*. 2007. Volume 19, Number 6: pp. 349 – 357
