# Supplementary material for: Context-driven mHealth solutions to improve maternal healthcare uptake in Ethiopia: Integrating stakeholder preferences and expert priorities using qualitative and nominal group techniques
Source: PLOS Digit Health. 2026 Nov 5;5(11):e0001817. doi: 10.1371/journal.pdig.0001817 (PMC13739138; doi:10.1371/journal.pdig.0001817)
Supplement: Supplementary file 3 [file pdig.0001817.s003.docx]

**S3 Appendix:** **KII guide for Key informants (HEWs, leaders of the female HDAs, and community leaders)**

**(English version)**

| General Information | |
| --- | --- |
| Interviewer’s name: ______________________________  KII’s site:  District____________________  Kebele____________________  Date፡ dd/mm/yy፡ __ __ /__ __/__ __ | Note taker’s name:  ___________________________  KII’s location:  ___________________________  Start time of the KII: ________:________  Completion time of the KII: ______:_____ |
| Key informants’ basic socio-demographic characteristics | |
| Age: _________  Sex:_________  Educational level፡_________  Work experiance: ______________ | |
| Introduction: | |
| Welcome! Today, we come together to gather your insights on preferred mHealth solution modalities such as SMS, calls, voice, videos, and apps. We also want to explore how these intervention modalities can be effectively delivered, whether through perinatal women alone, in combination with healthcare providers, social support groups, or community health workers. Additionally, we will discuss the timing for messages on weekdays (Sunrise to Midday, Midday to Sunset, or Sunset to Midnight), weekends (Sunrise to Midday, Midday to Sunset, or Sunset to Midnight), or based on gestational weeks to enhance maternal healthcare uptake. Your feedback is crucial in designing effective and culturally appropriate strategies.  Thank you for joining us! | |
| Detailed KII guide for key informants | |
| Questions | **Probing questions** |
| 1. What is the current status of maternal healthcare uptake during pregnancy, childbirth, and the postpartum period in your area? | - The coverage and utilization of ANC, SDC and PNC in the past five years - Trends in maternal healthcare-seeking behaviors over the past five years. - Which population are less likely to utilize these services? |
| 2. What are the common practices before, during, and after delivery in your area, and what are the underlying reasons for these practices? | - Cultural activity - How do people and mothers perceive about ANC, delievry, and PNC |
| 3. What are the main benefits that drives a mother to attend ANC, delivery in Health fascility and attend PNC in your area? | - What are the things or risks that you are most afraid of if you donot attend ANC, or deliver at Home, or have no PNC in your area? |
| 4. Where do you usually get maternal healthcare related information from? | - Consider the HEWs, HDA, Media, - Family information - What strategies do they to give you these information |
| 5. How would you explain the roles of family and community members in promoting maternal healthcare uptake during pregnancy, childbirth, and the postpartum period in your area? | - Male parents, mothers and fathers in law, TBAs, CHWs, community leaders - Which bodies support maternal healthcare uptake and which do not? What could be the reason behind this? |
| 6. What do you think are the barriers that women face in receiving appropriate maternal healthcare during pregnancy, childbirth, and the postpartum in your area? | - Individual level, health facilty level, community level, policy level - How do they overcome this barriers? |
| 7. What do you think are the factors that facilitate women's access to appropriate maternal healthcare during pregnancy, childbirth, and the postpartum in your area? | - Enabling factors, how do you look the facilitators in different levels including individual, health facilty, and community levels? |
| 8. Are you familiar with any mHealth interventions, such as SMS reminders, phone calls, voice messages, videos, or mobile apps, that are designed to enhance continuous access to appropriate maternal healthcare during pregnancy, childbirth, and the postpartum? | - If so, which ones are you familiar with or have interacted with? - Could you explain the scenario? How the implemntation and final resut was? |
| 9. How did you first hear about or learn about these mHealth interventions? | - Source of information about mHealth interventions |
| 10. How much do mothers in this area can use the above electronic messaging modalities? | - How many mothers have amobile phone? - Among those who have, how many can read short text messages? How many can can have a call conversation? How many can play videos? |
| 11. What do you think are the preferences and perceptions of specific mHealth intervention modalities in your area? a) SMS b) Phone calls, c) Voice messages, d) Videos e) Mobile applications f) Combined SMS with voice messages g) Combined SMS with video messages h) Combined SMS with direct voice calls | - How easy is it to use this modality? |
|  | - Do you feel this modality effective? |
|  | - Does it encourage interaction? |
|  | - Is this modality accessible to all users in your community? |
| 12. What do you think are the preferences and perceptions of specific mHealth intervention delivery approaches in your area? a) Through perinatal women only b) Through perinatal women and healthcare providers c) Through Perinatal women and social support groups d) Through perinatal women and CHWs e) Through Perinatal women, social supporters, and healthcare providers | - How easy is it to follow this delivery approaches? |
|  | - Do you feel this delivery approaches effective? |
|  | - Does it encourage interaction? |
|  | - Is this delivery approaches accessible to all users in your community? |
| 13. What do you think are the preferences and perceptions of specific mHealth intervention timing in your area? a) Weekdays (Sunrise to Midday, Midday to Sunset, Sunset to Midnight) b) weekends (Sunrise to Midday, Midday to Sunset, Sunset to Midnight) c) The day before the appointment based on GWks (Sunrise to Midday, Midday to Sunset, Sunset to Midnight) | - How easy is it to follow this timing? |
|  | - Do you feel this timing effective? |
|  | - Does it encourage interaction? |
|  | - Is this timing accessible to all users in your community? |
| 14. What challenges or barriers do you perceive might affect the implementation and effectiveness of mHealth interventions in your area?  - How do you think these could be addressed? | - Technical issues (e.g., connectivity, device access) - User engagement or motivation - Cultural appropriateness - How can we overcome this challenges (consider traing and others) |
| 15. Based on your experience and observation, what final recommendations or suggestions would you offer to help us design effective mHealth strategies that truly support maternal healthcare in your area? | - What key features do you think mHealth solutions need to effectively support mothers in your community? |
| Do you have any questions for me?  If not, I sincerely thank you for your time and active engagement in our discussion! | |
| ================== End =============== | |

**(Amharic Version)**

| አጠቃላይ መረጃ | | |
| --- | --- | --- |
| የጠያቂው ስም: ___________________________  ቃለ-መጠይቁ የተከናወነበት:  ወረዳ፡ _____________________  ቀበሌ፡ ______________________  ቃለ-መጠይቁ የተከናወነበት ቀን፡  ቀቀ/ወወ/ዓዓ፡__ __ /__ __/__ __ | | የማስታዋሻ ያዢ ስም:  ___________________________  ቃለ-መጠይቁ የተከናወነበት ጣቢያ:  ___________________________  ቃለ-መጠይቁ የተጀመረበት ሰዓት: _____:___  ቃለ-መጠይቁ የተጠናቀቀበት ሰዓት: ___:___ |
| የተሳታፊዎች መሠረታዊ መረጃ | | |
| እድሜ: ____________________________________________________  ታ:__________________________________________________________  የትምህርት ደረጃ፡______________________________________  ስራ / የስራ ሃላፊነት፡ ___________________________  የስራ ልምድ: _______________________________________ | | |
| መግቢያ: | | |
| እንኳን ደህና መጡ! ዛሬ፣ የእናቶችን ጤና አገልግሎት ተጠቃሚነትን ለማሻሻል የሚረዱ የሞባይል ጤና ዘዴዎች መካከል እንደ አጭር የፅሁፍ መልዕክቶች አገልግሎትን፣ የስልክ ቀጥታ ጥሪዎችን፣ የድምጽ መልዕክቶችን፣ የተንቀሳቃሽ ምስል መልዕክቶችን እና የሞባይል መተግበሪያዎችን በመሳሰሉት ዙሪያ የእርስዎን ግንዛቤ፣ ምርጫ፣ ተያያዥ ስጋቶችንና መልካም አጋጣሚዎችን ለመለየት ተገናኝተናል። እንዲሁም በእነዚህ የሞባይል ጤና ዘዴዎች የሚተላለፉ የእናቶች ጤናን የተመለከቱ የተመረጡ መረጃዎችና መልክቶች ለመለየትና ለነማን (ማለትም ለነፍሰጡርና ለወለዱ እናቶች ብቻ፣ ከጤና ባለሞያዎች ጋር፣ ከትዳር አጋራቸው ወይም ከመረቸው ሰዎች ጋር ወይስ ከማህበረሰብ ጤና ሰራተኞች ጋር) ቢላኩ የበለጠ ተቀባይነት አግኝተው የእናቶችን ጤና አገልግሎት ተጠቃሚነት ያሻሽላሉ የሚለውን መዳሰስ እንፈልጋለን። በተጨማሪም የእናቶች ጤናን የተመለከቱ የተመረጡ መረጃዎችንና መልክቶችን መቼ መቼ (ማልትም በሳምንቱ ቀናት (ከፀሐይ መውጫ እስከ እኩለ ቀን፣ ከቀትር እስከ ፀሐይ ስትጠልቅ፣ ወይም ከምትጠልቅበት እስከ እኩለ ሌሊት)፣ ቅዳሜና እሁድ (ከፀሐይ መውጫ እስከ እኩለ ቀን፣ እኩለ ቀን እስከ ፀሐይ ስትጠልቅ፣ ወይም ፀሐይ ከጠለቀች እስከ እኩለ ሌሊት)፣ ወይም የእርግዝና ሳምንታትን መሠረት በማድረግ) መላክ እንዳለብ እንወያይበታለን። ውጤታማ እና ለባህል ተስማሚ ስልቶችን በመንደፍ የእናቶችን ጤና አገልግሎት ተጠቃሚነት ለማሻሻል የእርስዎ ንቁ ተሳትፎና አስተያየት ወሳኝ ነው።  ለመሳተፍ ፈቃደኛ ስለሆኑ እናመሰግናለን! | | |
| የቃለ-መጠይቁ ዝርዝር | | |
| ጥያቄዎች | **ተከታይ ጥያቄዎች** | |
| 1. በአካባቢያችሁ በእርግዝና፣ በወሊድ እና በድህረ ወሊድ ወቅት የእናቶች ጤና አገልግሎትን ተጠቃሚነት አሁን ያለው ሁኔታ ምን ይመስላል? | - ባለፉት አምስት ዓመታት የቅድመ ወሊድ፣ የወሊድ እና የድህረ-ወሊድ ክትትል በጤና ተቋማት ያለው ሽፋን እና አጠቃቀም ምን ይመስላል? - ባለፉት አምስት ዓመታት የእናቶችን ጤና አገልግሎት ለመጠቀም የመፈለግ ባህሪ በእናቶችና ማህበረሰቡ ምን ይመስላል? - በአካባቢያችሁ በአብዛኛው የእናቶች የወሊድ ቦታ ምርጫ የት ነው? ቤት ወይስ ጤና ተቋም? ምክነያታቸውስ? - የትኞቹ የማህበረሰብ ክፍሎች ናቸው የእናቶች ጤና አገልግሎትን የመጠቀም እድላቸው አነስተኛ? | |
| 2. በአካባቢያችሁ ከወሊድ በፊት፣በወሊድ ጊዜ እና ከወለዱ በኋላ የተለመዱ ልማዶች ምንድን ናቸው? የእነዚህ ልማዶች መሰረታዊ ምክንያቶችስ ምንድን ናቸው? | - ባህላዊ ልማዶች እና እሳቤዎች ካሉ - የአካባቢዉ ማህበረሰብ እና እናቶች ስለ ቅድመ ወሊድ፣ በወሊድ ጊዜ እና በድህረ ወሊድ ወቅት በጤና ተቋም የሚደረግ ከትትልንና እንክብካቤን እንዴት ይገነዘባሉ? | |
| 3. በአካባቢያችሁ አንዲት እናት የቅድመ ወሊድ፣ የወሊድ እና ድህረ ወሊድ ክትትል በጤና ተቋም እንድትከታተል የሚያደርጓት ዋና ዋና ጥቅሞች ምንድን ናቸው? | - ቅድመ ወሊድ ክትትል ከሌለዎት፣ ወይም ቤት ውስጥ ከወለዱ፣ ወይም የድህረ ወሊድ ክትትል ከሌለዎት በጣም የሚፈሯቸው ነገሮች ወይም አደጋዎች ምንድን ናቸው? - ለእናት፣ ለተወለደው ህን፣ ለቤተሰብ፣ ለማህበረሰብ፣ ለሀገር የሚያስገኙት ጥቅሞች | |
| 4. ብዙውን ጊዜ የእናቶች ጤናን በተመለከተ መረጃን ከየት ያገኛሉ? | - የጤና ኤክስቴንሽን ሰራተኞችን፣ የጤና ልማት ሰራዊትን እና ሚዲያዎችን አስቡ። - በምን መልኩ ነው እነዚህ አካላት መረጃን እሚያደርሷችሁ? | |
| 5. በአካባቢያችሁ በእርግዝና፣ በወሊድ እና በድህረ ወሊድ ወቅት የእናቶች ጤና አገልግሎትን እንዲጠቀሙ በማገዝና በማሳወቅ ረገድ የቤተሰብ እና ማህበረሰብ አካላት ሚና እንዴት ይገለል? | - የትዳር አጋሮች፣ የአማች እናቶች እና አባቶች፣ ባህላዊ አዋላጆች፣ የማህበረሰብ ጤና ሰራተኞች እና የማህበረሰብ መሪዎች ሚና - የትኞቹ አካላት ድጋፍ ያረጋሉ? የትኞቹስ አትጠቀሙ ይላሉ? ምክንያቱስ ምን ይሆን? | |
| 6. በአካባቢያችሁ በእርግዝና፣ በወሊድ እና በድህረ ወሊድ ወቅት ሴቶች ተገቢውን የእናቶች ጤና አገልግሎት ለማግኘት የሚያጋጥሟቸው እንቅፋቶች ምንድን ናቸው ብለው ያስባሉ? | - በግለሰብ ደረጃ፣ በጤና ተቋም ደረጃ፣ በማህበረሰብ ደረጃ እና በፖሊሲ ደረጃ የሚያጋጥሟቸው እንቅፋቶች - እነዚህን እንቅፋቶች እንዴት ነው እሚያልፎቸው? | |
| 7. በአካባቢያችሁ በእርግዝና፣ በወሊድ እና በወሊድ ወቅት ሴቶች ተገቢውን የእናቶች ጤና አገልግሎት እንዲያገኙ የሚያግዙ ምቹ ሁኔታዎች ምን ምን ይመስላችኋል? | - ምቹና አበረታች ሁኔታዎችን፣ በግለሰብ፣ በጤና ተቋም፣ በማህበረሰብ እና በፖሊሲ ደረጃዎች እንዴት ይመለከቷቸዋል? | |
| 8. በእርግዝና፣ በወሊድ እና በድህረ-ወሊድ ጊዜ ተገቢውን የእናቶች ጤና አገልግሎቶችን ተደራሽነትን ለማሳደግ እንደ አጭር የፅሁፍ መልእክት አገልግሎት፣ የቀጥታ የስልክ ጥሪዎች፣ የድምጽ መልዕክቶች፣ የተንቀሳቃሽ ምስል መልዕክቶች ወይም እንደ ሞባይል መተግበሪያዎች ያሉ አገልግሎቶች አጋጥሞዎት ያውቃል? | - አጋጥሞዎት የሚያውቅ ከሆነ፣ የትኞቹን ያዉቃሉ፤ በየትኞቹስ ተሳታፊ ወይም ተጠቃሚ የመሆን እድል አጋጥሞዎት ነበር? - ምሳሌውን ቢያስረዱን፤ ተፈጻሚነቱ እና ዉጤቱ ምን ይመስላል? | |
| 9. ስለእነዚህ የሞባይል ጤና ዜዴዎች ለመጀመሪያ ጊዜ የሰሙት ወይም የተማሩት እንዴት ነው? | - ስለሞባይል ጤና ዜዴዎች የመረጃ ምንጭ | |
| 10. በዚህ አካባቢ ያሉ እናቶች ምን ያክል እነዚህን የጤና ዘዴዎች ባግባቡ መጠቀም ይችላሉ? | - ከህብረተሰቡ ዉስት ካሉ እናቶች መካከል ምን ያክሉ የሞባይል ስልክ አላችው? - ካላቸው ዉስጥስ ምን ያክሉ አጭር የጽሁፍ መልክትን ማንበበ ይችላሉ? ስንቶቹ ስልክ አንሰተው ማናገር ይችላሉ? ስንቶች ቪድዮ ማጫወት ይችላሉ? | |
| 11. በእርስዎ አካባቢ የእናቶች ጤና አገልግሎቶችን ተጠቃሚነትን ለመጨመር የሚረዱ የሞባይል ጤና ዜዴዎች መካከል የእናቶች እና የማህበረሰቡ ምርጫዎች የትኞቹ ናቸው ብለው ያስባሉ?   ሀ/ አጭር የፅሁፍ መልእክት አገልግሎት፣  ለ/ ቀጥታ የስልክ ጥሪዎችን በማድረግ፣  ሐ/ የድምጽ መልዕክቶችን በመላክ፣  መ/ በተንቀሳቃሽ ምስል መልዕክቶችን፣  ሠ/ በሞባይል መተግበሪያዎች መልክቶችንና  መረጃዎችን በማዘጋጀት በማጋራት፣  ረ/ አጭር የፅሁፍ መልእክትን ከድምጽ  መልዕክቶች ጋር በማቅቀናጀት በመላክ፣  ሰ/ አጭር የፅሁፍ መልእክትን ከተንቀሳቃሽ  ምስል መልዕክቶች ጋር በማቅቀናጀትና  ሸ/ አጭር የፅሁፍ መልእክትን ከስልክ  ጥሪዎች ጋር በማቀናጀት በመላክ | - ይህንን ዘዴ መጠቀም ምን ያህል ቀላል ነው? | |
|  | - ይህ ዘዴ ውጤታማነቱን እንዴት ያዩታል? | |
|  | - የሰዎችን መስተጋብር ያበረታታል? | |
|  | - ይህ ዘዴ በእርስዎ አካባቢ ማህበረሰብ ውስጥ ላሉ ሁሉም ተጠቃሚዎች ተደራሽ መሆን ይችላል? | |
| 12. አካባቢዎ በተመረጡ የሞባይል ጤና ዘዴዎች የሚተላለፉ የእናቶች ጤናን የተመለከቱ መረጃዎችንና መልዕክቶች ለነማን ቢላኩ የበለጠ ተቀባይነት አግኝተው የእናቶችን ጤና አገልግሎት ተጠቃሚነት ያሻሽላሉ ብለው ያስባሉ?   ሀ/ ለነፍሰጡርና ለወለዱ እናቶች ብቻ፣  ለ/ ለእናቶች እና ለጤና ባለሞያዎች፣  ሐ/ ለእናቶች እና ለትዳር አጋራቸው  ወይም ለመረቸው ሰዎች፣  መ/ ለእናቶች እና ለማህበረሰብ ጤና ሰራተኞች፣  ሠ/ ለእናቶች፣ ለጤና ባለሞያዎች እና  ለትዳር አጋራቸው ወይም  ለመረቸው ሰዎች | - ለነዚህ ሰዎች ወይም የህብረተሰብ ክፍሎች መረጃዎችንና መልዕክቶች መላክ ምን ያህል ቀላል ነው? | |
|  | - ለነዚህ ሰዎች ወይም የህብረተሰብ ክፍሎች መረጃዎችንና መልዕክቶችን መላክ የእናቶችን ጤና አገልግሎት ተጠቃሚነት ከማሻሻል አንር ውጤታማነቱን እንዴት ያዩታል? | |
|  | - የሰዎችን መስተጋብር ያበረታታል? | |
|  | - ይህ ዘዴ በእርስዎ አካባቢ ማህበረሰብ ውስጥ ላሉ ሁሉም ተጠቃሚዎች ተደራሽ መሆን ይችላል? | |
| 13. በአካባቢዎ የእናቶች ጤና አገልግሎቶችን ተጠቃሚነትን ለመጨመር የሚረዱ ከላይ በመረጣችኋቸው የሞባይል ጤና ዜዴዎች አማካኝነት ለመረጣችኋቸው ሰዎች ወይም የህብረተሰብ ክፍሎች መረጃዎችንና መልዕክቶችን መቼ መቼ መላክ አለባቸው ብለው ያስባሉ?   ሀ/ በሳምንቱ ቀናት (ከፀሐይ መውጫ እስከ  እኩለ ቀን፣ ከቀትር እስከ ፀሐይ ስትጠልቅ፣  ወይም ከምትጠልቅበት እስከ እኩለ ሌሊት)፣  ለ/ ቅዳሜና እሁድ (ከፀሐይ መውጫ እስከ  እኩለ ቀን፣ እኩለ ቀን እስከ ፀሐይ  ስትጠልቅ፣ ወይም ፀሐይ ከጠለቀች እስከ  እኩለ ሌሊት)፣  ሐ/ የእርግዝና ሳምንታትን መሠረት  በማድረግ (ከፀሐይ መውጫ እስከ እኩለ  ቀን፣ እኩለ ቀን እስከ ፀሐይ ስትጠልቅ፣  ወይም ፀሐይ ከጠለቀች እስከ እኩለ ሌሊት) | - ይህን ጊዜ መከተል ምን ያህል ቀላል ነው? | |
|  | - ይህን ጊዜ መከተል የእናቶችን ጤና አገልግሎት ተጠቃሚነት ከማሻሻል አንር ውጤታማነቱን እንዴት ያዩታል? | |
|  | - የሰዎችን መስተጋብር ያበረታታል? | |
|  | - ይህን ጊዜ መከተል በእርስዎ አካባቢ ማህበረሰብ ውስጥ ላሉ ሁሉም ተጠቃሚዎች ተደራሽ መሆን ይችላል? | |
| 14. በአካባቢዎ ያለውን የእናቶች ጤና አገልግሎት ተጠቃሚነትን ለማሻሻል የሞባይል ጤና ዜዴዎችን መጠቀም በትግበራ እና በውጤታማነት ላይ ተጽዕኖ ሊያሳድሩ የሚችሉት ተግዳሮቶች ወይም መሰናክሎች ምን ምን ይመስለዎታል?  - እንዴትስ ሊፈቱ ይችላሉ ብለው ያስባሉ? | - ቴክኒካዊ ጉዳዮች (ለምሳሌ፣ የአጠቃቀም እዉቀት፣ የስልክ አገልግሎት ተደራሽነት፣ የስልክ ቀፎ አለመኖር፣ የመብራትና ተያያዥ ችግሮች) - የተጠቃሚ ተሳትፎ ወይም ተነሳሽነት - ባህላዊ ተፅኖዎች - እነዚህ ችግሮች በምን መልኩ መቅረፍ ይቻላል? ሲልጠና መስጠት እንዲሁም ሌሎችን ያስቡ። | |
| 15. በመጨረሻ በእርስዎ ልምድ እና ምልከታ መሰረት የእናቶች ጤና አገልግሎት ተጠቃሚነትን ለማሻሻል የሚረዱ ውጤታማ የሞባይል ጤና ስልቶችን ለመንደፍ ምክረ ሃሳብ ካለዎት ያጋሩን? | - በአካባቢዎ ለሚገኙ እናቶች የእናቶች ጤና አገልግሎት ተጠቃሚነትን ለማሻሻል የሚዘጋጁ የሞባይል ጤና ዜዴዎች ምን ማሟላት ይኖርባቸዋል ብለው ያስባሉ? | |
| በእኔ በኩል የነበሩኝን ጥያቄዎች ጨርሻለሁ በእርስዎ በኩል የሚያነሷቸው ጥያቄዎች ወይም አስተያየቶች ካሉ ልቀበል?  ከሌለዎት፣ በውይይታችን ውድ ጊዜዎትን ስለሰጡኝና ስለነበርዎት ንቁ ተሳትፎ በእኔና በጥናት አባላት ስም ከልብ አመሰግናለው! | | |
| =============================== መጨረሻ ================================= | | |
