# Supplementary material for: Context-driven mHealth solutions to improve maternal healthcare uptake in Ethiopia: Integrating stakeholder preferences and expert priorities using qualitative and nominal group techniques
Source: PLOS Digit Health. 2026 Nov 5;5(11):e0001817. doi: 10.1371/journal.pdig.0001817 (PMC13739138; doi:10.1371/journal.pdig.0001817)
Supplement: Supplementary file 4 [file pdig.0001817.s004.docx]

**S4 Appendix:** **NGT guide for experts’ consensus (English version)**

| Basic socio-demographic characteristics | | | | |
| --- | --- | --- | --- | --- |
| Facilitator name name: ______________________ | | | NGT Expert’s code: ___________  Age: _________  Occupation: ______________  Educational level፡_________  Attention given for this interview:  ☐ 1. Maternal healthcare professionals  ☐ 2. Manager overseeing the maternity services  ☐ 3. Maternal health researchers  ☐ 4. Policymakers or executors  ☐ 5. Technology developers  ☐ 6. NGOs working on maternal health  ☐ 7. Behavioral science experts | |
| Date፡ dd/mm/yy፡ __ __ /__ __/__ __ | | | | Started time of the interview: ____:____  Completed time of interview: ____:____ |
| Introduction: | | | | |
| Welcome! Today, we come together to share insights from experts in clinical, behavioral, policy, technological, research, and NGO sectors working in maternal health. Our goal is to systematically identify and prioritize context-specific mHealth modalities, delivery methods, and timing to develop tailored interventions aimed at improving maternal healthcare uptake in southern Ethiopia. We are eager to hear your thoughts and recommendations based on the relevance, feasibility, and acceptability of various mHealth interventions, such as SMS, phone calls, voice messages, videos, and mobile applications in the case of Gamo zone, souther Ethiopia. Additionally, we want to explore how these interventions can be most effectively delivered, whether through perinatal women alone, in combination with healthcare providers, social support groups, or community health workers.  Timing is also a critical factor for the success of our mHealth interventions, and we would greatly appreciate your preferences and recommendations for messaging times in your area. Please indicate your preferred times for messages on weekdays (Sunrise to Midday, Midday to Sunset, Sunset to Midnight) and weekends (Sunrise to Midday, Midday to Sunset, Sunset to Midnight). Additionally, let us know your preferences for sending messages the day before an appointment, based on gestational weeks, within these same time slots.  Your feedback will help us better support expectant mothers in our community. Ultimately, we will identify the top three mHealth intervention modalities, delivery approaches, and message timing options based on the total scores provided by experts during the final rating process. We will then discuss these findings with the experts to reach a final consensus. Through our discussion today, we aim to foster collaboration and gather your invaluable insights to design effective mHealth strategies that genuinely support maternal healthcare in this region. Thank you for your participation in this important conversation! | | | | |
| NGT process | | | | |
| Steps | | **Activities** | | |
| 1 Preparation | - Ensure that all participants are informed about the purpose of the study and that they complete both a written consent form and demographic information | | | |
| 2 Introduction | - The facilitator will introduce the focus questions of the NGT sesions to the group and explain the NGT formats. | | | |
| 3 Presention | - The facilitator will present the current status of maternal healthcare uptake in Ethiopia, including access factors, barriers, facilitators, recent actions taken, and proposed mHealth solution modalities, approaches, and timing aimed at enhancing maternal healthcare. | | | |
| 4 Clarification | - Experts will have the opportunity to ask clarifying questions and suggest additional mHealth solution modalities, delivery approaches, and timing based on their expertise. | | | |
| 5 Individual preliminary rating (R1) | - Experts will independently rate the priority of mHealth solution modalities, approaches, and timing based on their assessments of reliability, feasibility, and acceptability within the local context. | | | |
| 6 Experts discussion of preliminary ratings | - Experts will share their perspectives and experiences with the mHealth solutions, explain the rationale behind their ratings, and address the challenges and enabling factors they anticipate related to tailoring interventions to the local context. | | | |
| 7 Individual final ratings (R2) | - Experts will provide their final individual ratings of the priority of mHealth solution modalities, approaches, and timing, and justify any significant deviations from the group consensus reached during the discussion | | | |
| 8 Closure | - A final experts' discussion will be held to communicate reporting timelines and conclude the workshop, followed by expressing gratitude to the participants. | | | |

**Annex V (a):** Forms for individual preliminary ratings (R1) and final ratings (R2) of context-specific mHealth modalities, delivery methods, and timing are provided. These ratings assess reliability, feasibility, and acceptability within the local context.

| mHealth solutions | | Relevance | | Feasible | | Acceptable | | Remark |
| --- | --- | --- | --- | --- | --- | --- | --- | --- |
|  |  | **R-1** | **R-2** | **R-1** | **R-2** | **R-1** | **R-2** |  |
| Modalities | SMS |  |  |  |  |  |  |  |
|  | Voice messages |  |  |  |  |  |  |  |
|  | Direct voice calls |  |  |  |  |  |  |  |
|  | Video messages |  |  |  |  |  |  |  |
|  | Mobile Apps |  |  |  |  |  |  |  |
|  | Combined SMS with voice messages |  |  |  |  |  |  |  |
|  | Combined SMS with video messages |  |  |  |  |  |  |  |
|  | Combined SMS with direct voice calls |  |  |  |  |  |  |  |
| Delivery approaches through | Only perinatal women |  |  |  |  |  |  |  |
|  | Perinatal women and healthcare providers |  |  |  |  |  |  |  |
|  | Perinatal women and social support groups |  |  |  |  |  |  |  |
|  | Perinatal women and CHWs |  |  |  |  |  |  |  |
|  | Perinatal women, social supporters, and healthcare providers |  |  |  |  |  |  |  |
| Timing (Days and times) | Weekday Sunrise to Midday |  |  |  |  |  |  |  |
|  | Weekend Sunrise to Midday |  |  |  |  |  |  |  |
|  | Weekday Midday to Sunset |  |  |  |  |  |  |  |
|  | Weekend Midday to Sunset |  |  |  |  |  |  |  |
|  | Weekday Sunset to Midnight |  |  |  |  |  |  |  |
|  | Weekend Sunset to Midnight |  |  |  |  |  |  |  |
|  | The day before the appointment, from sunrise to midday, based on their GWs |  |  |  |  |  |  |  |
|  | The day before the appointment, from Sunset to Midnight, based on their GWs |  |  |  |  |  |  |  |
|  | The day before the appointment, from Midday to Sunset, based on their GWs |  |  |  |  |  |  |  |

Based on your previous experience, write down additional mHealth modalities, delivery methods, and timing you think (if any) that would improve maternal healthcare uptake in our context?

*____________________________________________________________________________________________________________*

| Prioritization criteria | | Short description | Value |
| --- | --- | --- | --- |
| Relevance | Low | The proposed action has minimal influence on or is less important for increasing maternal healthcare service utilization. | 1 |
|  | Moderate | The proposed action supports the goal but has a less direct or smaller impact on increasing maternal healthcare service utilization. | 2 |
|  | High | The proposed action plays a crucial role in significantly increasing the utilization of maternal healthcare services. | 3 |
| Feasibility | Low | The proposed action is difficult to execute due to challenges related to resources, time, infrastructure, and environmental conditions. | 1 |
|  | Moderate | The proposed actions can be executed with appropriate adjustments, despite encountering some challenges related to available resources, time, infrastructure, and environmental conditions. | 2 |
|  | High | The proposed actions can be realistically executed within existing constraints, such as available resources, time, infrastructure, and environmental conditions, and they encounter few barriers. | 3 |
| Acceptability | Low | The proposed action is viewed as unsuitable, inconvenient, or problematic due to cultural differences, risks, barriers, or mistrust. | 1 |
|  | Moderate | The proposed action is somewhat positively received but faces barriers like cultural misalignment, accessibility issues, limited awareness, or misconceptions. | 2 |
|  | High | The proposed action is perceived as suitable, beneficial, and comfortable by most of the target population, supported by cultural compatibility, easy access, clear communication of benefits, community endorsement, and high trust and satisfaction. | 3 |

**Annex V (b):** Description of prioritization criteria for evaluating mHealth solution modalities, delivery approaches and message timing.
