# Supplementary material for: Context-driven mHealth solutions to improve maternal healthcare uptake in Ethiopia: Integrating stakeholder preferences and expert priorities using qualitative and nominal group techniques
Source: PLOS Digit Health. 2026 Nov 5;5(11):e0001817. doi: 10.1371/journal.pdig.0001817 (PMC13739138; doi:10.1371/journal.pdig.0001817)
Supplement: Supplementary file 5 [file pdig.0001817.s005.docx]

**S5 Appendix. Code-book**

| **Themes** | **Sub-themes** | **Codes** | **Code definitions** |
| --- | --- | --- | --- |
| Preferred mHealth modalities | Video-based education | Video | The participants reported that they preferred video-based educations to enhance their awareness of their health and the benefits of visiting health facilities. |
|  |  | Moving image | The participants informed that they preferred moving images to enhance their awareness of their health. |
|  | SMS combined with follow-up voice calls | Mobile message and voice | The participants described that they preferred mobile messages and voice calls for their appointment reminders and health information. |
|  |  | Texts with follow-up phone calls | The participants conveyed that they preferred receiving texts followed by phone calls for their appointment reminders and health information. |
|  |  | Texts and voice communications | The participants stated that they preferred receiving texts and voice calls for their appointment reminders and health information. |
|  | Direct voice calls alone | Phone calls | The participants described that they preferred only phone calls for their appointment reminders. |
|  |  | Voice calls | The participants reported that they preferred voice calls for their appointment reminders. |
|  |  | Two-way voice communication | The participants reported that they preferred two-way voice communication for their appointment reminders. |
| Preferred engagement approaches | Engaging perinatal women and their partners | Pregnant mothers and their partners | Participants stated that they preferred engaging pregnant mothers and their partners as a key personalized engagement approach for mHealth solutions. |
|  |  | Perinatal couples | The participants reported that they preferred engaging perinatal couples, particularly for appointment reminders. |
|  |  | Both mothers and fathers | The participants reported that they preferred engaging both mothers and fathers for maternal health information and appointment reminders. |
|  | Engaging perinatal women, CHWs, and designated community members | Collaborating with perinatal stakeholders | Participants informed that they preferred a combined strategy of collaborating with perinatal stakeholders in the development and dissemination of maternal health educational videos. |
|  |  | Engaging perinatal women and community representatives | Participants reported that they preferred engaging perinatal women and community representatives in the production and dissemination of maternal health educational videos. |
| Preferred timing of engagement | Sunrise to midday on the day before scheduled appointments based on gestational weeks | Morning before scheduled appointments | Participants suggested that reminders should be scheduled for the morning before scheduled appointments to help them prepare for their upcoming maternity visit. |
|  |  | Gestational week-based appointment preparation time | Participants reported that they preferred appointment reminders aligned with their gestational age. |
|  | Bi-weekly gatherings of perinatal women | Regular perinatal support meetings | Participants stated that they preferred integrating educational maternal health videos into perinatal women's regular biweekly support meetings, which take place on weekdays in easily accessible locations such as health posts or community centers. |
|  |  | Every other week maternal sessions | Participants reported that they preferred integrating educational maternal health videos into biweekly maternal sessions. |
| Perceived challenges to engagement | Limited access to devices | Lack of mobile phones | Participants reported that many perinatal women in their area lack personal mobile phones for appointment reminders and health information, potentially challenging equitable access to mHealth solutions. |
|  |  | Lack of compatible devices | Participants stated that some women have mobile phones that are not compatible with the mHealth platform, as they cannot store multiple SMS messages, resulting in notifications about insufficient storage after just a few messages. |
|  |  | No electricity | Participants reported that the lack of electricity, especially in rural areas, could potentially serve as a barrier hindering the consistent use of mHealth solutions. |
|  |  | Absence of charging facilities | Participants reported that even if there is electricity available, the lack of easily accessible charging facilities leading them to travel far from their area to charge their device, especially in rural areas, could potentially serve as a barrier hindering the consistent use of mHealth solutions. |
|  | Connectivity issues | No mobile network | Participants repeatedly noted that mobile network connectivity in many rural regions heavily depends on electricity, and since there is no electricity in this area, it poses a significant challenge. |
|  |  | On-off networks | Participants noted that even if mobile network connectivity is available, it is often unreliable in many rural regions, heavily dependent on electricity; when power outages occur, the entire network can go down, potentially severely hindering the dissemination of maternal health information and reminders through their phones. |
|  | Digital literacy concerns | Lack of familiarity | Participants reported that many perinatal women in their rural area lack familiarity with mobile devices, which may potentially challenge equitable access to mHealth solutions. |
|  |  | Lack of technical skills | Some participants expressed that their lack of technical skills, such as opening and reading messages on phones, could be confusing and challenging, often causing them to ignore important health information sent via messaging. |
