# Supplementary material for: Context-driven mHealth solutions to improve maternal healthcare uptake in Ethiopia: Integrating stakeholder preferences and expert priorities using qualitative and nominal group techniques
Source: PLOS Digit Health. 2026 Nov 5;5(11):e0001817. doi: 10.1371/journal.pdig.0001817 (PMC13739138; doi:10.1371/journal.pdig.0001817)
Supplement: Supplementary file 6 [file pdig.0001817.s006.docx]

| **A/ Round 1: Participant-level NGT ratings for** **mHealth solutions modalities** | | | | | | | | | | | | | | | | | | | | | | | | |
| --- | --- | --- | --- | --- | --- | --- | --- | --- | --- | --- | --- | --- | --- | --- | --- | --- | --- | --- | --- | --- | --- | --- | --- | --- |
| Participant (Expert)  Code | Short Message Service (SMS) | | | Voice Messages | | | Direct Voice Calls | | | Video Messages | | | Mobile Apps | | | Combined SMS with Voice Messages | | | Combined SMS with Video Messages | | | Combined SMS with Direct Voice Calls | | |
|  | R | F | A | R | F | A | R | F | A | R | F | A | R | F | A | R | F | A | R | F | A | R | F | A |
| E01 | 2 | 3 | 2 | 3 | 1 | 1 | 3 | 3 | 3 | 2 | 1 | 1 | 3 | 1 | 2 | 3 | 2 | 3 | 3 | 1 | 2 | 3 | 3 | 3 |
| E02 | 2 | 3 | 2 | 2 | 1 | 2 | 3 | 1 | 1 | 3 | 1 | 2 | 1 | 1 | 1 | 2 | 2 | 2 | 2 | 1 | 2 | 3 | 3 | 3 |
| E03 | 2 | 1 | 2 | 3 | 1 | 1 | 2 | 3 | 3 | 2 | 1 | 2 | 1 | 1 | 1 | 2 | 2 | 2 | 2 | 1 | 1 | 3 | 3 | 3 |
| E04 | 1 | 2 | 2 | 1 | 1 | 1 | 3 | 2 | 2 | 1 | 1 | 1 | 1 | 1 | 1 | 2 | 1 | 1 | 1 | 1 | 1 | 3 | 1 | 2 |
| E05 | 1 | 2 | 2 | 2 | 2 | 2 | 1 | 3 | 3 | 3 | 2 | 3 | 1 | 1 | 2 | 2 | 2 | 3 | 3 | 2 | 2 | 1 | 3 | 3 |
| E06 | 2 | 2 | 2 | 3 | 3 | 3 | 3 | 3 | 3 | 1 | 1 | 1 | 1 | 1 | 1 | 1 | 1 | 1 | 2 | 2 | 2 | 3 | 3 | 3 |
| E07 | 2 | 2 | 3 | 2 | 2 | 2 | 3 | 2 | 3 | 3 | 1 | 2 | 3 | 1 | 2 | 2 | 2 | 2 | 3 | 2 | 2 | 2 | 3 | 3 |
| E08 | 1 | 1 | 2 | 2 | 2 | 2 | 1 | 1 | 3 | 1 | 1 | 1 | 1 | 1 | 1 | 2 | 1 | 2 | 2 | 2 | 2 | 3 | 3 | 3 |
| E09 | 2 | 3 | 2 | 3 | 1 | 1 | 3 | 3 | 3 | 2 | 1 | 1 | 3 | 1 | 2 | 3 | 2 | 3 | 3 | 1 | 3 | 3 | 3 | 3 |
| E10 | 2 | 3 | 2 | 3 | 2 | 3 | 3 | 2 | 3 | 3 | 2 | 3 | 3 | 1 | 1 | 3 | 3 | 3 | 3 | 1 | 3 | 3 | 1 | 2 |
| E11 | 2 | 3 | 3 | 3 | 2 | 3 | 3 | 2 | 2 | 2 | 1 | 2 | 2 | 1 | 2 | 3 | 2 | 2 | 3 | 1 | 1 | 3 | 1 | 3 |
| E12 | 1 | 3 | 2 | 2 | 3 | 1 | 2 | 3 | 3 | 2 | 2 | 2 | 3 | 2 | 2 | 2 | 2 | 2 | 2 | 1 | 3 | 3 | 3 | 3 |
| E13 | 1 | 3 | 2 | 2 | 3 | 2 | 1 | 1 | 3 | 1 | 1 | 3 | 1 | 1 | 1 | 2 | 2 | 2 | 2 | 1 | 2 | 3 | 3 | 3 |
| E14 | 2 | 3 | 3 | 2 | 1 | 2 | 3 | 3 | 3 | 3 | 2 | 2 | 2 | 1 | 2 | 2 | 1 | 2 | 2 | 1 | 2 | 3 | 3 | 3 |

**S1 Table. De-identified participant-level NGT ratings for mHealth solutions modalities, engagement approaches, and timing preferences across relevance (R), feasibility (F), and acceptability (A) in Rounds 1 and 2.**

| **B/ Round 2: Participant-level NGT ratings for mHealth solutions modalities** | | | | | | | | | | | | | | | | | | | | | | | | |
| --- | --- | --- | --- | --- | --- | --- | --- | --- | --- | --- | --- | --- | --- | --- | --- | --- | --- | --- | --- | --- | --- | --- | --- | --- |
| Participant (Expert)  Code | Short Message Service (SMS) | | | Voice Messages | | | Direct Voice Calls | | | Video Messages | | | Mobile Apps | | | Combined SMS with Voice Messages | | | Combined SMS with Video Messages | | | Combined SMS with Direct Voice Calls | | |
|  | R | F | A | R | F | A | R | F | A | R | F | A | R | F | A | R | F | A | R | F | A | R | F | A |
| E01 | 2 | 3 | 2 | 2 | 1 | 2 | 3 | 3 | 3 | 1 | 1 | 1 | 3 | 2 | 1 | 2 | 2 | 3 | 3 | 2 | 2 | 3 | 3 | 3 |
| E02 | 2 | 3 | 2 | 3 | 1 | 2 | 3 | 2 | 3 | 3 | 1 | 3 | 1 | 1 | 1 | 2 | 1 | 2 | 3 | 1 | 2 | 3 | 1 | 3 |
| E03 | 2 | 1 | 1 | 2 | 1 | 2 | 3 | 3 | 3 | 2 | 2 | 2 | 1 | 1 | 1 | 3 | 2 | 2 | 2 | 2 | 2 | 3 | 3 | 3 |
| E04 | 3 | 1 | 1 | 2 | 1 | 1 | 3 | 2 | 3 | 1 | 1 | 2 | 1 | 1 | 2 | 2 | 1 | 3 | 2 | 1 | 1 | 3 | 2 | 3 |
| E05 | 1 | 2 | 2 | 2 | 3 | 2 | 1 | 2 | 1 | 2 | 3 | 3 | 1 | 1 | 1 | 2 | 2 | 2 | 3 | 1 | 3 | 2 | 3 | 3 |
| E06 | 2 | 3 | 2 | 3 | 2 | 2 | 3 | 3 | 3 | 2 | 2 | 2 | 2 | 1 | 2 | 2 | 2 | 2 | 2 | 2 | 2 | 3 | 3 | 2 |
| E07 | 2 | 2 | 3 | 3 | 2 | 3 | 3 | 2 | 3 | 3 | 1 | 2 | 3 | 1 | 1 | 3 | 1 | 2 | 3 | 1 | 3 | 3 | 1 | 3 |
| E08 | 1 | 1 | 1 | 2 | 2 | 2 | 3 | 1 | 3 | 1 | 1 | 1 | 1 | 1 | 1 | 2 | 2 | 2 | 2 | 1 | 2 | 3 | 3 | 3 |
| E09 | 1 | 2 | 2 | 3 | 2 | 2 | 1 | 3 | 3 | 2 | 1 | 2 | 3 | 1 | 2 | 3 | 3 | 3 | 3 | 2 | 2 | 3 | 3 | 3 |
| E10 | 2 | 3 | 1 | 3 | 2 | 3 | 3 | 3 | 3 | 3 | 3 | 3 | 3 | 2 | 1 | 3 | 2 | 3 | 3 | 2 | 2 | 3 | 3 | 3 |
| E11 | 2 | 3 | 3 | 3 | 2 | 3 | 3 | 2 | 3 | 2 | 2 | 2 | 2 | 1 | 2 | 3 | 1 | 2 | 3 | 2 | 2 | 3 | 3 | 3 |
| E12 | 1 | 3 | 3 | 3 | 1 | 1 | 3 | 3 | 3 | 1 | 3 | 3 | 1 | 1 | 1 | 2 | 2 | 3 | 3 | 2 | 3 | 3 | 3 | 3 |
| E13 | 1 | 3 | 1 | 2 | 3 | 2 | 1 | 1 | 3 | 1 | 1 | 3 | 1 | 1 | 1 | 2 | 2 | 2 | 2 | 1 | 2 | 3 | 3 | 3 |
| E14 | 1 | 3 | 3 | 2 | 1 | 1 | 3 | 3 | 1 | 3 | 2 | 2 | 2 | 1 | 1 | 3 | 1 | 2 | 2 | 2 | 2 | 2 | 3 | 3 |

| **C/ Round 1: Participant-level NGT ratings for engagement approaches** | | | | | | | | | | | | | | | |
| --- | --- | --- | --- | --- | --- | --- | --- | --- | --- | --- | --- | --- | --- | --- | --- |
| Participant (Expert)  Code | Only Perinatal Women | | | Perinatal Women and Healthcare Providers | | | Perinatal Women and Their Spouses, or Chosen Persons | | | Perinatal Women and Community Health Workers (CHWs) | | | Perinatal Women, Their Spouses, or Chosen Persons, and CHWs | | |
|  | R | F | A | R | F | A | R | F | A | R | F | A | R | F | A |
| E01 | 1 | 3 | 3 | 3 | 2 | 3 | 3 | 3 | 2 | 3 | 2 | 3 | 3 | 2 | 3 |
| E02 | 3 | 3 | 3 | 2 | 3 | 3 | 3 | 3 | 3 | 3 | 3 | 3 | 3 | 2 | 2 |
| E03 | 2 | 2 | 2 | 2 | 3 | 2 | 3 | 3 | 3 | 3 | 3 | 3 | 3 | 3 | 3 |
| E04 | 2 | 2 | 3 | 2 | 2 | 2 | 3 | 3 | 3 | 3 | 2 | 2 | 3 | 3 | 3 |
| E05 | 1 | 2 | 2 | 2 | 2 | 3 | 3 | 3 | 2 | 2 | 2 | 3 | 2 | 2 | 3 |
| E06 | 3 | 1 | 2 | 3 | 2 | 2 | 3 | 3 | 3 | 2 | 2 | 2 | 3 | 2 | 2 |
| E07 | 1 | 2 | 2 | 3 | 3 | 3 | 3 | 3 | 3 | 3 | 3 | 3 | 3 | 3 | 3 |
| E08 | 2 | 3 | 3 | 2 | 2 | 2 | 3 | 3 | 2 | 2 | 2 | 2 | 3 | 3 | 3 |
| E09 | 1 | 3 | 3 | 3 | 3 | 3 | 3 | 3 | 2 | 3 | 2 | 3 | 3 | 2 | 3 |
| E10 | 2 | 3 | 2 | 3 | 3 | 3 | 3 | 3 | 3 | 3 | 2 | 3 | 3 | 2 | 3 |
| E11 | 2 | 3 | 3 | 2 | 2 | 2 | 3 | 3 | 3 | 3 | 2 | 3 | 3 | 1 | 2 |
| E12 | 1 | 2 | 1 | 3 | 3 | 3 | 3 | 3 | 3 | 3 | 3 | 3 | 3 | 3 | 3 |
| E13 | 1 | 1 | 2 | 2 | 2 | 2 | 2 | 2 | 3 | 2 | 3 | 3 | 3 | 3 | 3 |
| E14 | 1 | 3 | 1 | 2 | 2 | 2 | 3 | 3 | 3 | 3 | 3 | 3 | 3 | 1 | 1 |

| **D/ Round 2: Participant-level NGT ratings for engagement approaches** | | | | | | | | | | | | | | | |
| --- | --- | --- | --- | --- | --- | --- | --- | --- | --- | --- | --- | --- | --- | --- | --- |
| Participant (Expert)  Code | Only Perinatal Women | | | Perinatal Women and Healthcare Providers | | | Perinatal Women and Their Spouses, or Chosen Persons | | | Perinatal Women and Community Health Workers (CHWs) | | | Perinatal Women, Their Spouses, or Chosen Persons, and CHWs | | |
|  | R | F | A | R | F | A | R | F | A | R | F | A | R | F | A |
| E01 | 1 | 3 | 3 | 3 | 2 | 3 | 3 | 3 | 2 | 3 | 3 | 3 | 3 | 2 | 3 |
| E02 | 2 | 3 | 3 | 2 | 3 | 2 | 3 | 3 | 3 | 3 | 3 | 3 | 2 | 2 | 2 |
| E03 | 2 | 2 | 2 | 3 | 3 | 3 | 3 | 3 | 3 | 2 | 2 | 2 | 2 | 2 | 3 |
| E04 | 2 | 2 | 3 | 3 | 3 | 3 | 3 | 3 | 3 | 2 | 2 | 2 | 3 | 3 | 3 |
| E05 | 1 | 3 | 2 | 2 | 2 | 3 | 3 | 3 | 2 | 2 | 2 | 2 | 2 | 3 | 3 |
| E06 | 2 | 3 | 3 | 3 | 2 | 2 | 3 | 3 | 3 | 3 | 2 | 3 | 2 | 1 | 2 |
| E07 | 1 | 2 | 3 | 3 | 3 | 3 | 3 | 3 | 3 | 3 | 3 | 3 | 3 | 3 | 3 |
| E08 | 2 | 2 | 2 | 2 | 2 | 2 | 2 | 2 | 2 | 2 | 2 | 2 | 3 | 3 | 3 |
| E09 | 1 | 3 | 3 | 2 | 3 | 3 | 3 | 2 | 2 | 3 | 2 | 3 | 3 | 2 | 3 |
| E10 | 1 | 3 | 3 | 2 | 3 | 3 | 3 | 3 | 3 | 3 | 3 | 3 | 3 | 3 | 3 |
| E11 | 2 | 3 | 3 | 2 | 2 | 2 | 3 | 3 | 3 | 3 | 2 | 3 | 3 | 2 | 2 |
| E12 | 3 | 3 | 3 | 3 | 3 | 3 | 3 | 2 | 1 | 3 | 2 | 1 | 3 | 2 | 2 |
| E13 | 1 | 1 | 2 | 2 | 2 | 2 | 2 | 2 | 3 | 2 | 3 | 3 | 3 | 3 | 3 |
| E14 | 1 | 3 | 2 | 2 | 2 | 3 | 3 | 3 | 3 | 3 | 3 | 3 | 2 | 3 | 3 |

| **E/ Round 1: Participant-level NGT ratings for timing of mHealth solutions** | | | | | | | | | | | | | | | | | | | | | | | | | | | |
| --- | --- | --- | --- | --- | --- | --- | --- | --- | --- | --- | --- | --- | --- | --- | --- | --- | --- | --- | --- | --- | --- | --- | --- | --- | --- | --- | --- |
| Participant (Expert)  Code | Weekday Sunrise to Midday | | | Weekend Sunrise to Midday | | | Weekday Midday to Sunset | | | Weekend Midday to Sunset | | | Weekday Sunset to Midnight | | | Weekend Sunset to Midnight | | | The Day Before the Appointment, from Sunrise to Midday | | | The Day Before the Appointment, from Midday to Sunset | | | The Day Before the Appointment, from Sunset to Midnight | | |
|  | R | F | A | R | F | A | R | F | A | R | F | A | R | F | A | R | F | A | R | F | A | R | F | A | R | F | A |
| E01 | 2 | 3 | 1 | 1 | 3 | 3 | 2 | 3 | 3 | 3 | 3 | 3 | 2 | 3 | 1 | 1 | 1 | 1 | 3 | 3 | 3 | 3 | 3 | 3 | 3 | 3 | 3 |
| E02 | 3 | 3 | 3 | 3 | 3 | 3 | 3 | 2 | 2 | 2 | 2 | 2 | 1 | 2 | 1 | 1 | 2 | 1 | 3 | 3 | 3 | 3 | 2 | 2 | 2 | 2 | 1 |
| E03 | 3 | 3 | 3 | 1 | 1 | 1 | 2 | 2 | 2 | 1 | 1 | 1 | 1 | 1 | 1 | 1 | 1 | 1 | 3 | 3 | 3 | 1 | 1 | 2 | 2 | 1 | 3 |
| E04 | 2 | 2 | 2 | 1 | 1 | 1 | 2 | 2 | 2 | 2 | 2 | 2 | 2 | 2 | 1 | 2 | 2 | 2 | 3 | 3 | 3 | 3 | 3 | 2 | 1 | 2 | 2 |
| E05 | 1 | 3 | 2 | 1 | 3 | 3 | 3 | 2 | 2 | 1 | 1 | 1 | 1 | 1 | 1 | 1 | 1 | 1 | 2 | 3 | 3 | 1 | 3 | 3 | 2 | 2 | 3 |
| E06 | 3 | 2 | 3 | 2 | 3 | 3 | 2 | 3 | 3 | 2 | 2 | 2 | 2 | 1 | 1 | 1 | 1 | 1 | 3 | 3 | 3 | 3 | 1 | 2 | 3 | 1 | 1 |
| E07 | 2 | 2 | 2 | 1 | 1 | 1 | 3 | 3 | 3 | 2 | 2 | 2 | 1 | 1 | 1 | 2 | 3 | 2 | 2 | 3 | 2 | 2 | 3 | 2 | 2 | 3 | 2 |
| E08 | 2 | 3 | 2 | 2 | 2 | 2 | 3 | 2 | 2 | 3 | 2 | 2 | 3 | 2 | 3 | 2 | 2 | 2 | 3 | 2 | 3 | 3 | 3 | 3 | 2 | 3 | 3 |
| E09 | 2 | 3 | 1 | 1 | 3 | 3 | 2 | 3 | 1 | 1 | 3 | 3 | 1 | 3 | 1 | 1 | 3 | 1 | 3 | 3 | 3 | 3 | 3 | 2 | 3 | 3 | 3 |
| E10 | 2 | 3 | 1 | 2 | 3 | 3 | 3 | 2 | 3 | 2 | 2 | 3 | 3 | 3 | 3 | 2 | 2 | 2 | 3 | 3 | 2 | 1 | 2 | 3 | 3 | 3 | 2 |
| E11 | 3 | 2 | 3 | 2 | 3 | 2 | 2 | 3 | 3 | 3 | 3 | 3 | 2 | 1 | 1 | 2 | 1 | 1 | 3 | 3 | 3 | 1 | 2 | 2 | 1 | 3 | 3 |
| E12 | 2 | 2 | 2 | 1 | 3 | 3 | 3 | 3 | 3 | 2 | 2 | 2 | 3 | 3 | 3 | 1 | 2 | 1 | 3 | 3 | 3 | 3 | 1 | 3 | 3 | 1 | 1 |
| E13 | 3 | 1 | 1 | 2 | 1 | 1 | 2 | 2 | 2 | 1 | 1 | 2 | 2 | 2 | 2 | 2 | 1 | 1 | 3 | 3 | 2 | 3 | 2 | 2 | 3 | 1 | 3 |
| E14 | 1 | 3 | 2 | 2 | 2 | 3 | 2 | 3 | 2 | 3 | 3 | 3 | 1 | 2 | 1 | 3 | 2 | 2 | 2 | 3 | 3 | 2 | 2 | 2 | 3 | 3 | 3 |

| **F/ Round 2: Participant-level NGT ratings for timing of mHealth solutions** | | | | | | | | | | | | | | | | | | | | | | | | | | | |
| --- | --- | --- | --- | --- | --- | --- | --- | --- | --- | --- | --- | --- | --- | --- | --- | --- | --- | --- | --- | --- | --- | --- | --- | --- | --- | --- | --- |
| Participant (Expert)  Code | Weekday Sunrise to Midday | | | Weekend Sunrise to Midday | | | Weekday Midday to Sunset | | | Weekend Midday to Sunset | | | Weekday Sunset to Midnight | | | Weekend Sunset to Midnight | | | The Day Before the Appointment, from Sunrise to Midday | | | The Day Before the Appointment, from Midday to Sunset | | | The Day Before the Appointment, from Sunset to Midnight | | |
|  | R | F | A | R | F | A | R | F | A | R | F | A | R | F | A | R | F | A | R | F | A | R | F | A | R | F | A |
| E01 | 2 | 3 | 1 | 1 | 3 | 3 | 2 | 3 | 3 | 3 | 3 | 3 | 2 | 3 | 3 | 1 | 1 | 1 | 3 | 3 | 3 | 2 | 3 | 2 | 3 | 3 | 3 |
| E02 | 3 | 3 | 3 | 2 | 3 | 2 | 2 | 2 | 2 | 1 | 2 | 2 | 1 | 2 | 1 | 1 | 1 | 1 | 3 | 3 | 3 | 1 | 2 | 2 | 3 | 2 | 3 |
| E03 | 3 | 3 | 3 | 2 | 2 | 2 | 2 | 1 | 1 | 2 | 2 | 2 | 1 | 1 | 1 | 1 | 1 | 1 | 3 | 2 | 2 | 1 | 2 | 2 | 2 | 2 | 2 |
| E04 | 2 | 2 | 3 | 2 | 2 | 2 | 3 | 2 | 2 | 2 | 2 | 2 | 1 | 1 | 1 | 2 | 2 | 2 | 3 | 3 | 3 | 2 | 2 | 2 | 3 | 3 | 3 |
| E05 | 1 | 2 | 2 | 1 | 2 | 2 | 2 | 2 | 2 | 1 | 1 | 1 | 1 | 1 | 1 | 1 | 1 | 1 | 3 | 3 | 3 | 1 | 3 | 2 | 1 | 1 | 1 |
| E06 | 3 | 2 | 3 | 3 | 2 | 2 | 1 | 1 | 1 | 2 | 2 | 2 | 1 | 1 | 1 | 1 | 1 | 1 | 3 | 3 | 3 | 2 | 1 | 1 | 3 | 2 | 3 |
| E07 | 3 | 3 | 3 | 1 | 1 | 1 | 3 | 3 | 3 | 2 | 2 | 2 | 1 | 1 | 1 | 1 | 1 | 1 | 3 | 3 | 3 | 2 | 3 | 2 | 3 | 3 | 3 |
| E08 | 2 | 2 | 2 | 1 | 1 | 1 | 2 | 2 | 2 | 2 | 2 | 2 | 2 | 2 | 3 | 2 | 2 | 2 | 2 | 2 | 3 | 3 | 3 | 3 | 2 | 3 | 3 |
| E09 | 1 | 3 | 1 | 2 | 2 | 2 | 2 | 2 | 1 | 2 | 3 | 3 | 2 | 2 | 2 | 1 | 2 | 2 | 3 | 2 | 3 | 3 | 2 | 3 | 3 | 2 | 3 |
| E10 | 3 | 3 | 1 | 2 | 3 | 3 | 3 | 2 | 2 | 3 | 3 | 3 | 2 | 2 | 2 | 3 | 3 | 3 | 3 | 3 | 3 | 3 | 3 | 3 | 2 | 2 | 1 |
| E11 | 3 | 2 | 3 | 2 | 2 | 2 | 2 | 3 | 3 | 2 | 2 | 3 | 2 | 1 | 1 | 1 | 1 | 1 | 3 | 3 | 3 | 2 | 1 | 1 | 3 | 3 | 3 |
| E12 | 2 | 2 | 2 | 2 | 2 | 2 | 3 | 3 | 3 | 2 | 2 | 2 | 1 | 2 | 1 | 1 | 3 | 1 | 3 | 3 | 3 | 2 | 2 | 2 | 2 | 2 | 1 |
| E13 | 3 | 1 | 1 | 1 | 1 | 1 | 1 | 2 | 1 | 1 | 2 | 2 | 1 | 2 | 2 | 1 | 2 | 1 | 3 | 3 | 3 | 3 | 3 | 3 | 3 | 3 | 3 |
| E14 | 2 | 3 | 2 | 2 | 3 | 3 | 2 | 3 | 2 | 3 | 2 | 3 | 2 | 3 | 1 | 3 | 2 | 1 | 3 | 3 | 3 | 3 | 3 | 1 | 3 | 3 | 3 |
