# Supplementary material for: Context-driven mHealth solutions to improve maternal healthcare uptake in Ethiopia: Integrating stakeholder preferences and expert priorities using qualitative and nominal group techniques
Source: PLOS Digit Health. 2026 Nov 5;5(11):e0001817. doi: 10.1371/journal.pdig.0001817 (PMC13739138; doi:10.1371/journal.pdig.0001817)
Supplement: Supplementary file 7 [file pdig.0001817.s007.docx]

**S2 Table. Sensitivity analysis of mHealth modalities, engagement approaches, and timings prioritization rankings across alternative aggregation methods.**

| Priority domain | Option | Primary weighting (25% R1 + 75% R2):  Score/126 (Rank) | R2 only:  Score/126 (Rank) | Equal weighting (50% R1+ 50% R2):  Score/126 (Rank) | Median-based: Score/9 (Rank) | Rank change / Stability summary |
| --- | --- | --- | --- | --- | --- | --- |
| mHealth modalities | SMS | 84.3 (6) | 83.0 (6) | 85.5 (4*) | 6.5 (3) | Ranking shifts: Rank 6 in primary, and R2-only; shifts to Rank 4 in equal weighting and 3 in median-based |
|  | Voice messages | 86.3 (4) | 87.0 (5) | 85.5 (4*) | 6.0 (4*) | Minor shift: Rank 4 in primary, equal weightings, and median-based; shifts to Rank 5 in R2-only |
|  | Direct voice calls | 106.3 (2) | 107.0 (2) | 105.5 (2) | 8.0 (2) | Stable (Rank 2 across all methods) |
|  | Video messages | 79.8 (7) | 82.0 (7) | 77.5 (7) | 6.0 (4*) | Ranking shifts: Rank 7 in primary, R2-only, and equal weightings; shifts to Rank 4 in median-based |
|  | Mobile Apps | 59.8 (8) | 59.0 (8) | 60.5 (8) | 4.0 (8) | Stable (Rank 8 across all methods) |
|  | Combined SMS with voice messages | 89.8 (3) | 91.0 (3) | 88.5 (3) | 6.0 (4*) | Minor shift: Rank 3 in primary, R2-only, and equal weightings; shifts to Rank 4 in median-based |
|  | Combined SMS with video messages | 85.8 (5) | 88.0 (4) | 83.5 (6) | 6.0 (4*) | Minor shift: Rank 5 in Primary; shifts to Rank 4 in R2-only and median-based, and Rank 6 in equal weighting |
|  | Combined SMS with direct voice calls | 117.3 (1) | 118.0 (1) | 116.5 (1) | 9.0 (1) | Stable (Rank 1 across all methods) |
| Engagement approaches | Only perinatal women | 93.3 (5) | 95.0 (5) | 91.5 (5) | 7.0 (4*) | Minor shift: Rank 5 in primary, R2-only, and equal weightings; shifts to Rank 4 in median-based |
|  | Perinatal women and healthcare providers | 105.3 (4) | 106.0 (4) | 104.5 (4) | 7.0 (4*) | Stable (Rank 4 across all methods) |
|  | Perinatal women and their spouses, or chosen persons | 115.5 (1) | 114.0 (1) | 117.0 (1) | 9.0 (1) | Stable (Rank 1 across all methods) |
|  | Perinatal women and CHWs | 108.0 (3) | 107.0 (3) | 109.0 (3) | 8.0 (2*) | Minor shift: Rank 3 in primary, R2-only, and equal weightings; shifts to Rank 2 in median-based |
|  | Perinatal women, their spouses, or chosen persons, and CHWs | 109.3 (2) | 109.0 (2) | 109.5 (2) | 8.0 (2*) | Stable (Rank 2 across all methods) |
| Timing of mHealth solutions | Weekday Sunrise to Midday | 96.3 (3) | 97.0 (3) | 95.5 (3*) | 6.0 (6*) | Ranking shifts: Rank 3 in primary, R2-only, and equal weightings; shifts to Rank 6 in median-based |
|  | Weekend Sunrise to Midday | 82.3 (7) | 81.0 (7) | 83.5 (7) | 6.5 (5) | Minor shift: Rank 7 in primary, R2-only, and equal weightings; shifts to Rank 5 in median-based |
|  | Weekday Midday to Sunset | 92.3 (5) | 89.0 (6) | 95.5 (3*) | 7.0 (3*) | Minor shift: Rank 5 in primary weighting; shifts to Rank 6 in R2-only, Rank 3 in equal weighting and median-based |
|  | Weekend Midday to Sunset | 89.5 (6) | 90.0 (5) | 89.0 (6) | 6.0 (6*) | Minor shift: Rank 6 in primary, equal weightings, and median-based; shifts to Rank 5 under R2-only |
|  | Weekday Sunset to Midnight | 67.0 (8) | 65.0 (8) | 69.0 (8) | 4.0 (8*) | Stable (Rank 8 across all methods) |
|  | Weekend Sunset to Midnight | 62.8 (9) | 62.0 (9) | 63.5 (9) | 4.0 (8*) | Minor shift: Rank 9 in primary, R2-only, and equal weightings; shifts to Rank 8 in median-based |
|  | The day before the appointment, from sunrise to midday | 120.5 (1) | 121.0 (1) | 120.0 (1) | 9.0 (1) | Stable (Rank 1 across all methods) |
|  | The day before the appointment, from Midday to Sunset | 93.0 (4) | 92.0 (4) | 94.0 (5) | 7.0 (3*) | Minor shift: Rank 4 in primary, and R2-only; shifts to Rank 5 in equal weightings, and Rank 3 in median-based |
|  | The day before the appointment, from Sunset to Midnight | 103.0 (2) | 105.0 (2) | 101.0 (2) | 8.0 (2) | Stable (Rank 2 across all methods) |

*SMS = Short Message Services; CHWs = Community Health Workers;* *R1 = Round 1; R2 = Round 2. Scores represent summed relevance, feasibility, and acceptability ratings across the 14 experts, evaluated out of a maximum of 126 points (14 experts × 3 evaluation criteria × 3 maximum score per criterion). For the primary analysis, scores were weighted 25% for Round 1 and 75% for Round 2. For the equal-weighting analysis, each round contributed 50%; Median-based scores were calculated by summing relevance, feasibility, and acceptability ratings (R + F + A; range: 3–9) for each expert in each round, with the median calculated across 28 participant-round observations (14 experts × 2 rounds) for each modality; * Indicates tied scores share the same rank; “Minor shift” denotes a change of ≤2 ranking positions across the alternative aggregation methods, and larger changes are reported as “ranking shifts.”*
